# Genome-wide replication landscape of *Candida glabrata*

**DOI:** 10.1186/s12915-015-0177-6

**Published:** 2015-09-02

**Authors:** Stéphane Descorps-Declère, Cyril Saguez, Axel Cournac, Martial Marbouty, Thomas Rolland, Laurence Ma, Christiane Bouchier, Ivan Moszer, Bernard Dujon, Romain Koszul, Guy-Franck Richard

**Affiliations:** Institut Pasteur, Unité de Génétique Moléculaire des Levures, Département Génomes & Génétique, F-75015 Paris, France; CNRS, UMR3525, F-75015 Paris, France; Sorbonne Universités, UPMC Univ Paris 06, 4 Place Jussieu, 75252, Paris, Cedex 05 France; Institut Pasteur, Center of Bioinformatics, Biostatistics and Integrative Biology (C3BI), F-75015 Paris, France; Institut Pasteur, Groupe Régulation Spatiale des Génomes, Département Génomes & Génétique, F-75015 Paris, France; Present address: Institut Pasteur, Unité de Génétique Humaine et Fonctions Cognitives, Département des Neurosciences, F-75015 Paris, France; Institut Pasteur, Plate-forme Génomique, Département Génomes & Génétique, F-75015 Paris, France; Present address: Plate-forme Bio-informatique/Biostatistique, Institut de Neurosciences Translationnelles IHU-A-ICM, Hôpital Pitié-Salpêtrière, 47–83 bd de l’Hôpital, 75561, Paris, Cedex 13 France

**Keywords:** 3C, ACS, ARS, *Candida glabrata*, Chromosome organization, Megasatellites, Replication, Yeast

## Abstract

**Background:**

The opportunistic pathogen *Candida glabrata* is a member of the Saccharomycetaceae yeasts. Like its close relative *Saccharomyces cerevisiae,* it underwent a whole-genome duplication followed by an extensive loss of genes. Its genome contains a large number of very long tandem repeats, called megasatellites. In order to determine the whole replication program of the *C. glabrata* genome and its general chromosomal organization, we used deep-sequencing and chromosome conformation capture experiments.

**Results:**

We identified 253 replication fork origins, genome wide. Centromeres, *HML* and *HMR* loci, and most histone genes are replicated early, whereas natural chromosomal breakpoints are located in late-replicating regions. In addition, 275 autonomously replicating sequences (ARS) were identified during ARS-capture experiments, and their relative fitness was determined during growth competition. Analysis of ARSs allowed us to identify a 17-bp consensus, similar to the *S. cerevisiae* ARS consensus sequence but slightly more constrained. Megasatellites are not in close proximity to replication origins or termini. Using chromosome conformation capture, we also show that early origins tend to cluster whereas non-subtelomeric megasatellites do not cluster in the yeast nucleus.

**Conclusions:**

Despite a shorter cell cycle, the *C. glabrata* replication program shares unexpected striking similarities to *S. cerevisiae*, in spite of their large evolutionary distance and the presence of highly repetitive large tandem repeats in *C. glabrata*. No correlation could be found between the replication program and megasatellites, suggesting that their formation and propagation might not be directly caused by replication fork initiation or termination.

**Electronic supplementary material:**

The online version of this article (doi:10.1186/s12915-015-0177-6) contains supplementary material, which is available to authorized users.

## Background

In the present work, replication properties of the *C. glabrata* genome were determined using high-throughput sequencing and chromosome conformation capture (3C). Replication origins were mapped and early firing origins were found to be clustered in the nucleus, suggesting some level of organization in the temporal replication program. When replication origins and termination sites were analyzed, no evidence for an enrichment of large tandem repeats near replication origins or termination sites was found. However, chromosomal rearrangements tended to be more frequent in late-replicating regions, suggesting that these regions may be more prone to breakage and rearrangement during replication.

Genome replication is the preliminary and essential step to the reproduction of any living organism. To achieve that goal, a complex dedicated machinery has been set up and propagated throughout evolution. It consists of interdependent *cis*- and *trans*-acting factors, respectively replication origins and a protein machinery involved in the catalytic process of replicating DNA. The identification of replication origins in eukaryotes started with the discovery that some autonomous DNA sequences were necessary and sufficient to replicate a plasmid in the model yeast *Saccharomyces cerevisiae* [1, 2]. Since then, genome-wide catalogs of autonomously replicating sequences (ARS) have been established in *S. cerevisiae* [3, 4], *Lachancea kluyveri* [5], *Lachancea waltii* [6], *Kluyveromyces lactis* [7], *Candida albicans* [8], and *Pichia pastoris* [9]. However, not all ARSs function as bona fide chromosomal replication origins [10] and a great deal of energy has been devoted, for the last 15 years, to the systematic identification and characterization of active chromosomal origins in yeast species, using different genomic approaches. Microarray hybridization of genomic DNA probes extracted at different stages during the cell cycle identified chromosomal origins and replication termination sites in *S. cerevisiae* [11–13], *Schizosaccharomyces pombe* [14], and *Lachancea kluyveri* [15]. Other methods used to characterize replication origins include immunoprecipitation of origin-bound pre-replication complexes, purification of nascent-strand DNA, capture and identification of replication bubbles (reviewed in [16, 17]), characterization of single-stranded DNA regions [18], or comparative genomics between closely related species [19]. More recently, an approach using deep sequencing was developed. Synchronized G1 yeast cells were released through the cell cycle, and DNA was collected at different predefined intervals and sequenced to a high enough coverage to distinguish between unreplicated DNA (sequence coverage = 1) and replicated DNA (sequence coverage = 2). This powerful approach was applied to three Taphrinomycotina species, *Schizosaccharomyces pombe*, *Schizosaccharomyces octoporus*, and *Schizosaccharomyces japonicus* [20]; four *Saccharomyces sensu stricto* species; and one hybrid [21]. It also confirmed origins that were previously identified in *Lachancea kluyveri* by microarray analysis [15]. Here, we applied a related approach to characterize chromosomal replication origins and termination sites in *C. glabrata*.

*C. glabrata* belongs to the Nakaseomyces and its genome underwent the same whole-genome duplication that occurred in *S. cerevisiae* ancestors [22]. It is an opportunistic pathogen involved in epithelial candidiasis and bloodstream infections in immunocompromised patients [23]. Adhesion of *C. glabrata* to human epithelial cells is mediated by a family of calcium-dependent adhesins, called *EPA* genes [24], more abundant in pathogenic species of this clade [25].

In addition to *EPA* genes, *C. glabrata* encodes 44 genes containing large tandem repeats called megasatellites [26]. These megasatellites are made of large motifs (ca. 100–400 bp long), tandemly repeated from three to 32 times, located in genes potentially involved in cellular adhesion to host cells [27]. At the present time, there is no clear explanation either for the presence of so many megasatellites in the *C. glabrata* genome, or for the mechanism leading to their formation and subsequent propagation, despite extensive phylogenetic analyses [28, 29].

The *C. glabrata* genome exhibits frequent genomic polymorphisms, including copy-number variations, chromosomal translocations, and formation of neochromosomes, some of them occurring within close proximity to megasatellites [30, 31]. Determining the complete replication program of *C. glabrata* may therefore improve our understanding of relationships between megasatellites, genomic rearrangements, and S phase replication.

## Results

### Identification of chromosomal replication origins and correlations to characterized genetic features

**Fig. 1:**
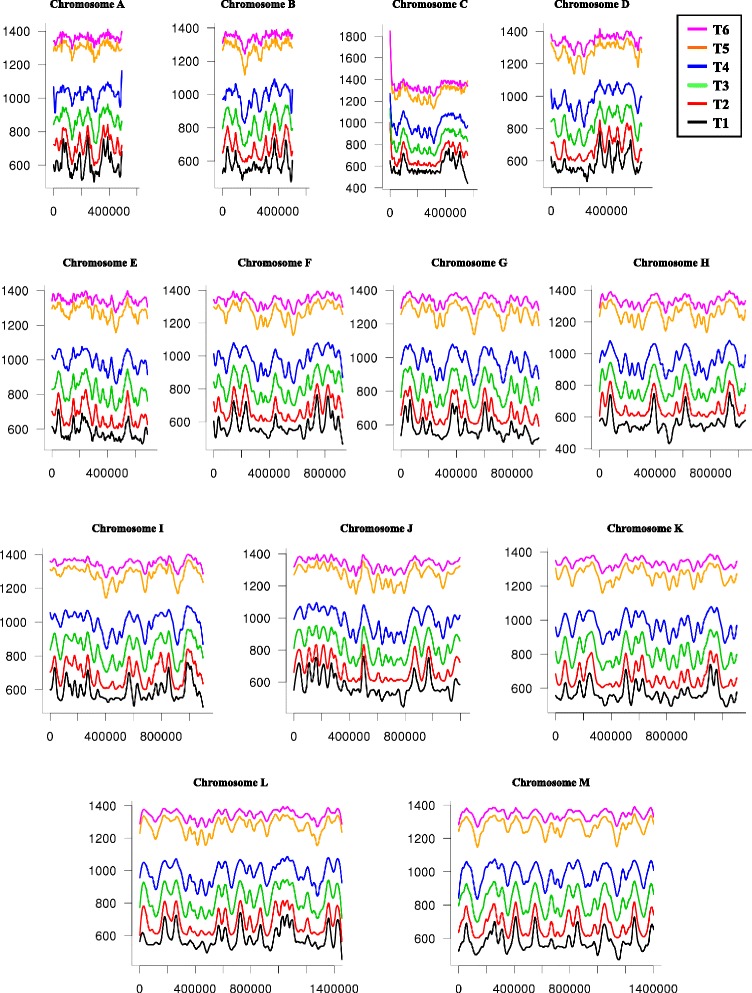
Sequence coverage during S-phase time course. The sequence coverage at each time point (T1 to T6) for each of the 13 chromosomes, is shown. For each nucleotide (on the *x axis*) at every time point, relative sequence coverage is plotted (on the *y axis*) as the ratio of Tn coverage over T0 coverage (see “Methods”). Early replication origins are visible as peaks in the first time points, and gradually disappear when regions between origins are being replicated

**Fig. 2:**
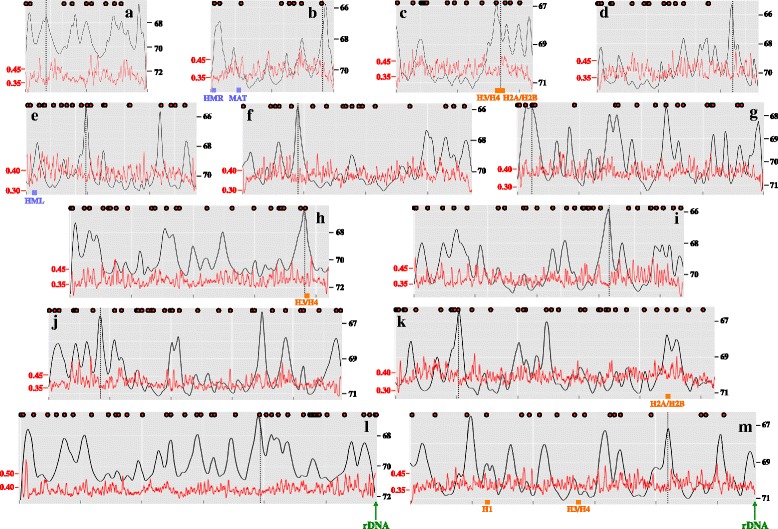
Replication landscape of *C. glabrata* chromosomes. For each of the 13 chromosomes (labeled from **a** to **m**), the T_50_ of each nucleotide is shown as a *black line*, peaks correspond to replication origins, and valleys to termini. Post-elutriation times, after release in fresh medium, are indicated on the *right* side of each graph (in minutes). The GC content of each chromosome is drawn as a *red line*, with GC % indicated on the *left* side of each graph (5 kb sliding windows by 500 bp steps). The distance between two *gray vertical bars* under each graph is 250 kb. *Dotted vertical lines* represent centromere positions. *MAT*, *HML*, and *HMR* loci are shown as *blue boxes*, at their location as determined by Muller et al. [76]. Histone genes are shown as *orange boxes*. Subtelomeric locations of both rDNA tandem arrays are indicated by *green arrows* on chromosomes L and M. Autonomously replicating sequences positions are indicated above each graph as *red dots*

**Fig. 3:**
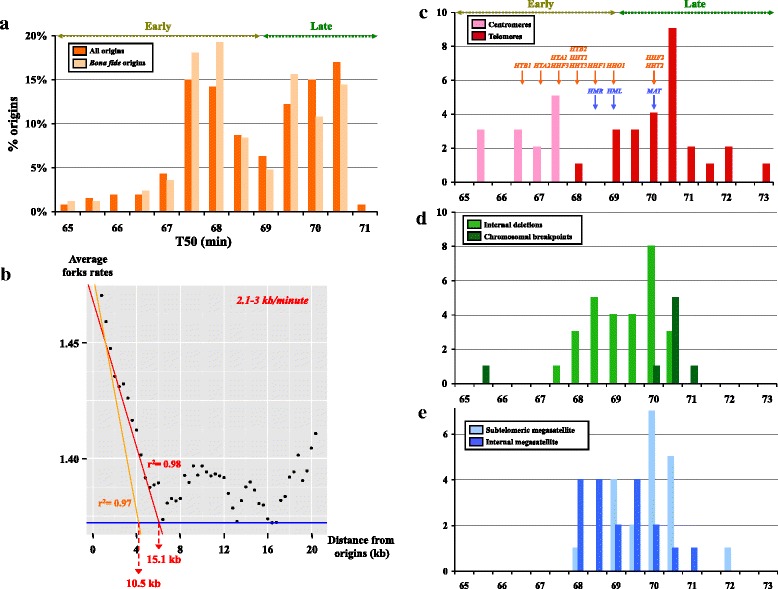
Replication timing of chromosomal features. **a** Distribution of early and late origins, according to their T_50_. The percentage of each class is represented on the *y axis*. Each interval corresponds to 30 s. Early and late origins are defined according to the observed distribution of the two populations of origins, origins firing before 69 min being labeled as early. The distribution of bona fide origins is shown in *light orange*, and is not statistically different from the whole distribution. **b** Determination of the average initial replication fork speed. Average fork rates, shown as *black dots*, are plotted every 400 bp according to their distance from early origins (only bona fide early origins were considered). The *orange line* corresponds to the linear regression of the first 2 kb. The *red line* corresponds to the linear regression of the first 5 kb. Correlation coefficients (r^2^) are indicated near each line. The *blue line* corresponds to the baseline at which no replication occurs. Intersection of each linear regression with the baseline indicates the amount of DNA replicated within a 5-min time frame (10.5 kb or 15.1 kb) for an average fork speed between 2.1 kb/min and 3 kb/min. **c** Distribution of replication timing of centromeres and telomeres, according to their T_50_. Intervals to which mating-type cassettes belong are indicated by *blue arrows* and histone genes by *orange arrows*. Note that “Early” and “Late” replication time frames correspond to those defined in a. **d** Distribution of replication timing of internal chromosomal deletions and chromosomal breakpoints found in translocations, according to their T_50_. **e** Distribution of replication timing of subtelomeric and internal megasatellites, according to their T_50_

It was previously shown that *C. glabrata* does not respond to a-mating or α-mating pheromones [32]. It was therefore impossible to use this approach to obtain a homogeneous population of G1 cells, as can be done with other yeast species. Centrifugal elutriation was therefore chosen to isolate small, unbudded, G1 daughter cells from asynchronously growing populations (“Methods”; [33]). Approximately 5 × 10^9^ cells were elutriated and resuspended in fresh yeast extract peptone dextrose (YPD) medium (T0 time point). After 55 min, the first time point (T1) was collected and following samples were collected every 5 min for 90 min. Fluorescence-activated cell sorting (FACS) analysis showed that approximately 15 % of elutriated cells had already partially replicated their genome because a peak corresponding to G2 DNA content was already visible at T0 (Additional file 1). This suggests that *C. glabrata* enters S phase before starting to make any bud, and that early origins probably fire soon after mitosis exit. Single read sequences corresponding to DNA extracted from time points T0 to T6 were subsequently mapped to the CBS138 reference genome. Coverage for each nucleotide, at every time point, was plotted as compared to the coverage of the same nucleotide at T0 (Fig. 1). Several coverage peaks are already visible at T1, corresponding to activated origins. Coverage was monitored throughout S phase, until the DNA content of the whole chromosome eventually doubled. For each nucleotide of the genome, a T_50_ value, corresponding to the time at which this nucleotide was replicated in 50 % of the cells, was calculated and plotted to identify replication origins (Fig. 2, black lines). Altogether, 253 origins were identified, spaced 31–64 kilobases (kb) from each other (average: 49 kb, Table 1). There was no visible correlation between GC content (Fig. 2, red lines) and replication curves (Fig. 2, black lines), origins being distributed independently of any apparent composition bias. When comparing timings of origin activation, two equal-sized populations were found, corresponding to two bursts of origins firing (Fig. 3a, Additional File 2).

Average genome-wide replication fork speeds were determined for early origins and were plotted according to their distance from origins (Fig. 3b). As expected, coverage tended to decrease with distance, because regions located far from an origin are replicated later. The average fork speed was found by linear extrapolation of fork rates (orange or red lines) to the point at which fork rates become null (horizontal blue line). It was found to be 10.5–15.1 kb, corresponding to an average fork speed of 2.1–3 kb/min, in the range calculated for *S. cerevisiae* (2.3 kb/min) [12], *L. kluyveri* (2.9 kb/min) [15], and *S. pombe* (2.8 kb/min) [34].

All centromeres were replicated in the first burst of activation, by early origins. Telomeres were replicated in late S phase following the second burst of origin firing (Fig. 3c), although one telomere (chromosome C, left arm) was replicated earlier. However, given sequencing incompleteness of *C. glabrata* subtelomeric regions, it is most likely that this telomere was actually replicated later on. Centromeres were always located near the very first origin firing on each chromosome, except for chromosome A (Fig. 2). Note that both donor mating-type cassettes, *HML* and *HMR*, were replicated at approximately the same time and before the mating-type locus *MAT*. Histone genes were mostly replicated early during S phase (see “Discussion”).

Replication timings of known internal chromosomal deletions and chromosomal breakpoints found in translocations [30, 31] were determined. Interestingly, chromosomal breakpoints were located more often than randomly expected in late-replicating regions (seven occurrences out of eight breakpoints, Fig. 3d), suggesting that they are more prone to either breakage or unfaithful repair. This was not true for internal deletions, which were as often located in early as in late-replicating regions (Fisher exact test *p* value = 0.1364).

Replication timing of megasatellites presented a bias for late regions (Fisher exact test *p* value = 0.03146), but when subtelomeric megasatellites were discarded, the remainder did not exhibit any bias regarding replication timing (Fig. 3e). One thousand independent random sets of non-subtelomeric megasatellites were simulated. Their average distance to the closest replication origin was calculated to be 15.8 ± 0.2 kb, not significantly different from the observed distance to origins (13.2 ± 4.1 kb) or to replication termini (13.1 ± 4.4 kb). Therefore, megasatellites do not appear to be linked to the close proximity of origins or termini.

### Different replication timings of chromosome C arms

**Fig. 4:**
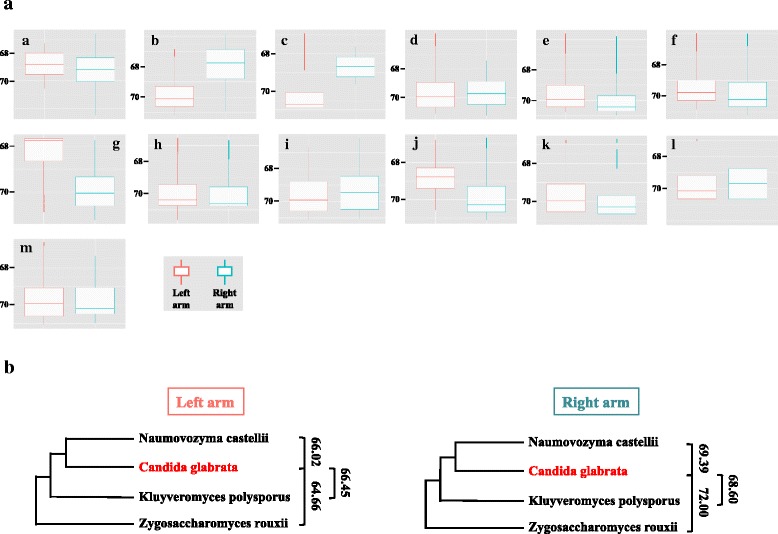
Replication timing of chromosomal arms. **a** For each chromosome arm, T_50_ are represented as boxplots. *Red*: left chromosome arm values; *blue*: right chromosome arm values. **b** Phylogeny of C-left and right arm genes. Average Z-scores of distances between *C. glabrata* genes and closely related yeast species are shown for each chromosome C arm. None of these distances was significantly different from the other (*t*-test *p*-values of C left Z-scores Nc vs Kp, 0.93; Nc vs Zr, 0.79; *t*-test *p*-values of C right Z-scores Nc vs Kp, 0.90; Nc vs Zr, 0.70; significance threshold 0.05). *Kp Kluyveromyces polysporus*, *Nc Naumovozyma castellii*, *Zr Zygosaccharomyces rouxii*

In order to detect a possible bias toward early or late replication of a chromosome arm, similar to what has been described for *L. kluyveri* [15], T_50_ were plotted for each chromosome arm separately. No significant difference was observed between left and right arms, except for three chromosomes, B, C, and G (Fig. 4a). Chromosomes B and G were acrocentric (Fig. 2). Therefore, the observed difference in arm replication timing for these two chromosomes was probably due to the quasi-absence of origins in one arm. For chromosome C, however, the centromere was located three-quarters along the chromosome length, and its right arm replicated earlier. No difference in GC content was detected between left and right arms (Fig. 2), as compared to what was observed for *L. kluyveri* chromosome C left arm [15], presenting a 10 % GC enrichment and a much earlier replication pattern. Because hybridization between yeast species and large DNA introgressions are very common among yeasts [35, 36], it is possible that the two arms of *C. glabrata* chromosome C had different origins, explaining their different replication patterns. All chromosome C proteins were extracted and compared to *Naumovozyma castellii*, *Kluyveromyces polysporus*, and *Zygosaccharomyces rouxii* complete proteomes. These three species were chosen because they are the closest sequenced species outside the *C. glabrata* clade [22]. Proteome comparisons and clustering (“Methods” and Fig. 4b), showed no detectable evidence that *C. glabrata* chromosome C left and right arm proteins come from different phylogenetic origins. In support of this conclusion, mapping of ancestral centromeres by Gordon et al. [37] showed that *C. glabrata CEN3* is an ancestral centromere and synteny is conserved across it, a result hardly compatible with chromosomal fusion of two different chromosomes. It must be concluded that the difference in replication timing observed for these two chromosomal arms is therefore not due to different phylogenetic origins.

### Whole-genome identification of ARSs

ARSs were originally defined as DNA sequences providing extrachromosomal maintenance of plasmids in *S. cerevisiae* [2, 10]. Since then, ARSs have been characterized in many yeast species [3–9]. In order to identify *C. glabrata* ARSs, a complete genomic library of small DNA fragments (ca. 200 bp) was cloned in a plasmid containing the *C. glabrata URA3* gene but lacking a replication origin (“Methods”). In a first experiment, the library was transformed in a strain in which *URA3* was entirely deleted (strain HM100 [32]) and 1,558 transformant colonies were obtained. Plasmidic DNA was extracted from these colonies and deep-sequenced. Using this approach, 180 different DNA fragments were mapped to the *C. glabrata* genome, ranging in size from 35 to 493 nucleotides (nt) (mean = 240 ± 14 nt). In a second independent experiment, 2,616 transformant colonies were obtained and 227 fragments were mapped, ranging from 36 to 480 nt (mean = 215 ±13 nt), 132 fragments being common to both experiments. Altogether, 275 DNA fragments able to propagate a plasmid in *C. glabrata* were identified. Given that 132 fragments were common to both sets of ARSs, we estimated that the *C. glabrata* genome should contain a total of 310–320 ARSs. During the second experiment, the library was also transformed in *S. cerevisiae* cells and 274 fragments were mapped (mean size = 205 ± 9 nt).

**Fig. 5:**
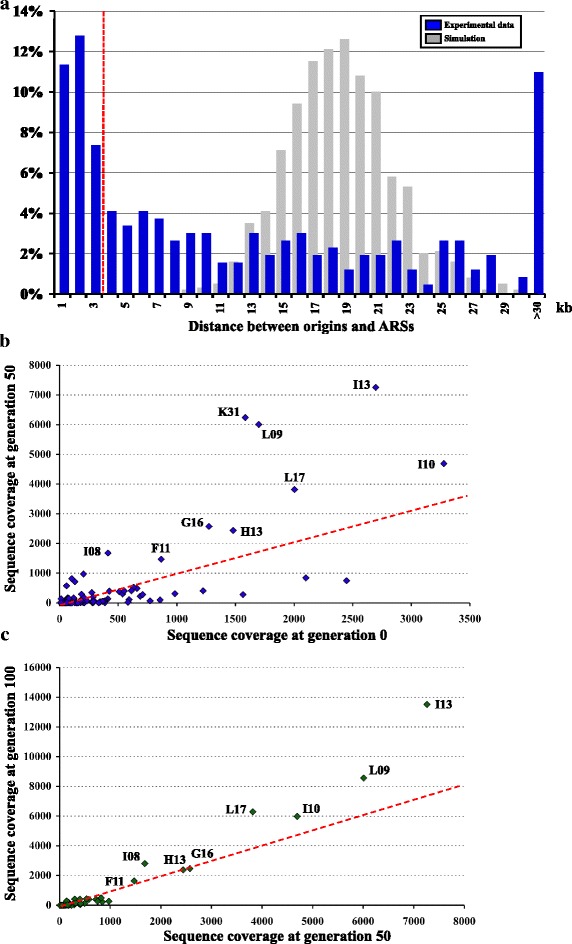
ARS capture and fitnesses. **a** Distribution of distances between ARSs and replication origins. The observed distribution is shown in blue. The simulation of 1,000 independent experiments is shown in gray (see text). The dotted red line corresponds to the 3-kb distance limit that was chosen to define bona fide origins. **b** Coverage of each ARS at G50 (*y axis*) as compared to G0 (*x axis*). The *dotted red line* corresponds to a coverage ratio 1/1. ARSs with the highest G50 coverage are labeled. **c** Same as b, but for G100 coverage of each ARS (*y axis*), as compared to G50 coverage (*x axis*). *ARS* autonomously replicating sequence

ARSs were numbered on each chromosome, according to their position, from the left to the right telomere. ARS positions were compared to replication origins, and the distance between each ARS and the closest replication origin was computed. About half of ARSs (150/275, 55 %) were found within 10 kb of a replication origin (Fig. 5a, blue bars ), with a majority of those within 3 kb (83/150). The set of origins close to these 83 ARSs were considered as bona fide origins and were used for subsequent statistical analyses. As a control, 1,000 random sets of 275 DNA fragments (random ARSs) were generated genome wide. The same number of random ARSs as compared to real ARSs was generated per chromosome, and their distance to the closest origin was calculated. The average result of these 1,000 simulations followed a normal distribution (mean = 18.5 kb, sigma = 3.3 kb) and was strikingly different from the observed distribution (Fig. 5a, gray bars).

In order to estimate replication peak resolution, we amplified, cloned in a *URA3*-plasmid, and transformed in *C. glabrata*, eight regions of 4 kb surrounding origins in which no ARS was detected. As a positive control, a bona fide origin on chromosome G containing an ARS (ARS_G11) was also cloned and transformed. Growth of the transformed cells was estimated after 1 and 4 days, in liquid culture, at 30 °C, in synthetic complete (SC-Ura) synthetic medium. The positive control as well as two plasmids showed normal growth in these conditions. Two plasmids showed slow growth and would not have been detected in the ARS capture experiment, and four plasmids did not grow at all (Additional file 3). Therefore, only two out of eight 4-kb regions surrounding a replication peak were able to propagate a plasmid, showing that peak resolution is above 2 kb, a conclusion already suggested by the average distance found between peaks and ARSs (Fig. 5a). This was confirmed by a second independent replication peak determination, in which average distance between bona fide origins found in both experiments was 6.9 ± 2.4 kb (Additional file 4).

**Fig. 6:**
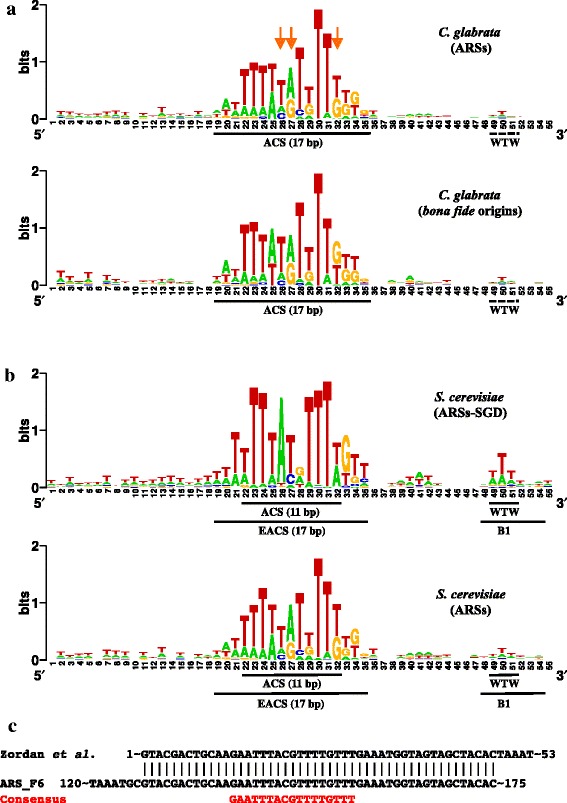
ARS consensus sequences. **a** *C. glabrata* ACS determined from the whole set of 275 ARSs (*top*), or from the 83 bona fide origins (*bottom*). Boundaries of the element are clearly visible in the latter case. The three positions differing from the *S. cerevisiae* ACS are shown by *orange arrows*. The WTW trinucleotide was barely detected. **b** *S. cerevisiae* ACS determined from the set of 337 known ARSs listed in the *Saccharomyces Genome Database* (*SGD*) [70] (*top*), or from the 274 ARSs identified here (*bottom*). Boundaries of the canonical ACS [10] or of the extended ACS (*EACS*) are indicated, as well as the B1 box and WTW trinucleotide [38]. Note that although the ACS identified in the ARS capture experiment was closer to the *C. glabrata* ACS than to the *S. cerevisiae* canonical ACS, the requirement for the B1 box was greater in *S. cerevisiae* than in *C. glabrata*. **c** Alignment of ARS_F6 with the ARS identified by Zordan et al. [46], showing 48 bp in common between the two sequences. The ACS is shown in *red. ACS* ARS consensus sequence, *ARS* autonomously replicating sequence

Using the motif finder GIMSAN, we identified a 17-bp A/T-rich ARS consensus sequence (ACS) common to the 275 DNA fragments selected (Fig. 6a). This ACS is similar to the core *S. cerevisiae* 11-bp ACS [10] and the 17-bp extended ACS [38], except for slight differences on three positions. When only the set of 83 bona fide origins was considered, a nearly identical motif was detected, with boundaries clearly visible (Fig. 6a). The same analysis was performed with ARSs extracted from *S. cerevisiae*. The motif detected was closer to the *C. glabrata* ACS than to the canonical *S. cerevisiae* ACS (Fig. 5b). However, when the B1 sequence was considered, particularly the WTW trinucleotide [38], it was found to be more conserved in ARSs replicating in *S. cerevisiae* than in ARSs replicating in *C. glabrata*. Altogether these observations strongly suggest that the requirement for the B1 box is weaker in *C. glabrata*, and that the information necessary to initiate replication is mostly contained in the 17 bp ACS, in which some positions are more constrained than in the *S. cerevisiae* ACS.

### rDNA replication

In *S. cerevisiae*, rDNA tandem repeat units are carried by chromosome XII, which contains 100–200 tandem units per genome [39, 40]. One ARS is present in each rDNA repeat unit (ARS1200), and is located between the 18S and the 5S rDNA genes (Additional file 5). In *C. glabrata*, rDNA tandem repeat units are found on two different chromosomes, L and M, both in subtelomeric positions [41]. Two ARSs were detected in *S. cerevisiae*, ARS_L38 and ARS_L39, both falling in the same area as ARS1200, between the 18S and 5S genes (Additional file 5). Coverage of the ARS library before transformation was determined to be 776 ± 11 for ARS_L38. Given that the library mean coverage was 9× (Additional file 6), we concluded that this region was represented in 86 ± 1 copies in the genome. This figures the average number of rDNA tandem repeat units in the *C. glabrata* genome, corresponding to 40–50 repeat units per chromosome. This number was slightly lower than in *S. cerevisiae*, but budding yeast contains an exceptionally high number of rDNA copies as compared to other fungi [39].

### ARS fitness

In order to determine replication efficiency of each ARS, a sub-fraction (10 %) of the cell suspension, corresponding to a total 1,558 pooled colonies collected in the first ARS capture experiment, was grown for 100 additional generations in a medium selective for plasmid maintenance. DNA was extracted from cells after 50 generations (G50), or after 100 generations (G100), and deep-sequenced. Altogether, 109 independent ARSs were identified at G50, and 77 at G100 (compared to 180 ARSs at the beginning of the experiment, G0). All ARSs found at G100 were already present at G50 and all ARSs present at G50 were already present at G0 (Additional file 7). Sequencing coverage of each ARS at each generation time was considered to represent replication efficiency, because efficient ARSs should be more often represented than others. G50 coverage was plotted against G0 coverage and showed that 25 ARSs were more represented after 50 generations, nine of them exhibiting more than 1,000-fold coverage (Fig. 5b). Similarly, G100 coverage was plotted against G50 coverage, and showed that 11 ARSs were more represented after an additional 50 generations, eight out of the former nine ARSs still exhibiting more than 1,000-fold coverage. The only ARS that was no longer represented at G100 among the former nine was ARS_K31 (Fig. 5c). Mean sizes of mapped DNA fragments were 221 ± 15 nt and 235 ± 18 nt for G50 and G100 respectively (as compared to 195 ± 12 nt for G0). This suggests that during the course of this competition experiment, plasmids that replicated more efficiently also contained a slightly larger insert size, probably to more easily accommodate the fixation of ORC and MCM complexes [42].

Absolute fitness of a given genotype was defined as the ratio of the number of individuals with that genotype at generation N + 1 to the number of individuals with the same genotype at generation N. In replication competition experiments, efficacy of a given ARS at a given generation was considered to equal the sequence coverage of that ARS. Hence, absolute fitness of each ARS was determined as [(coverage at generation N + 50)/(coverage at generation N)]/50. This was determined for the first 50 generations, as well as for the following 50 generations, and averaged. At G50, absolute fitness ranged from 0 (the ARS is not represented anymore after 50 generations) to 0.2196 for ARS_K06. Similarly, G100 absolute fitness ranged from 0 to 0.0433 for ARS_K06, and the average absolute fitness for G50 + G100 ranged from 0 to 0.1314 for ARS_K06. Relative fitness was calculated by giving the value 1 to the ARS with the highest average absolute fitness, other ARSs being given proportional relative fitnesses between 0 and 1 (Additional file 7).

Table 2 summarizes locations and properties of the nine ARSs exhibiting the highest coverage at G50 and G100 (as shown in Fig. 5b,c). There was no obvious correlation with distances to the closest origin, only four ARSs out of nine being located less than 2 kb from an origin. However, they all exhibited a relative fitness ≥20 % (only 16 out of 180 ARSs showed a relative fitness >20 %). Hence, any of these nine ARSs would be a good choice for an efficient multicopy plasmid replicator in *C. glabrata*.

**Table 1:** General features of the *C. glabrata* replication landscape

Chromosome	Size (kb)	Origins	kb/Ori	Terminations	kb/Ter
A	491	16	31	15	41
B	502	17	30	16	42
C	559	13	43	13	43
D	652	15	43	14	38
E	688	16	43	17	34
F	927	21	44	20	49
G	992	19	52	18	52
H	1050	20	53	20	48
I	1100	20	55	19	58
J	1195	22	54	21	54
K	1303	26	50	26	57
L	1456^a^	26	56	25	58
M	1403^a^	22	64	22	56
Total	12318	253	49	248	50

^a^Not including rDNA repeat arrays

*kb* kilobase pairs

**Table 2:** Location and characteristics of the nine most efficient ARSs of the *C. glabrata* genome

ARS	Start	End	Size (bp)	Closest origin	Ori-ARS (nt)	T50	Relative fitness (%)
F11	471,720	472,032	312	479,174	7,454	70.49	21.4
G16	770,672	770,894	222	792,365	21,693	70.35	22.7
H13	433,274	433,562	288	416,726	16,548	69.67	20.0
I08	343,823	344,156	333	342,422	1,401	69.57	43.8
I10	448,861	449,108	247	450,097	1,236	69.98	20.6
I13	566,761	567,059	298	565,468	1,293	68.37	34.7
K31	1,152,867	1,153,097	230	1,150,055	2,812	68.02	30.0
L09	359,511	359,818	307	360,585	1,074	70.50	37.8
L16	725,573	725,932	359	666,139	6,176	70.44	27.1

*ARS* autonomously replicating sequence, *bp* base pairs, *nt* nucleotides

### Chromosomal organization

**Fig. 7:**
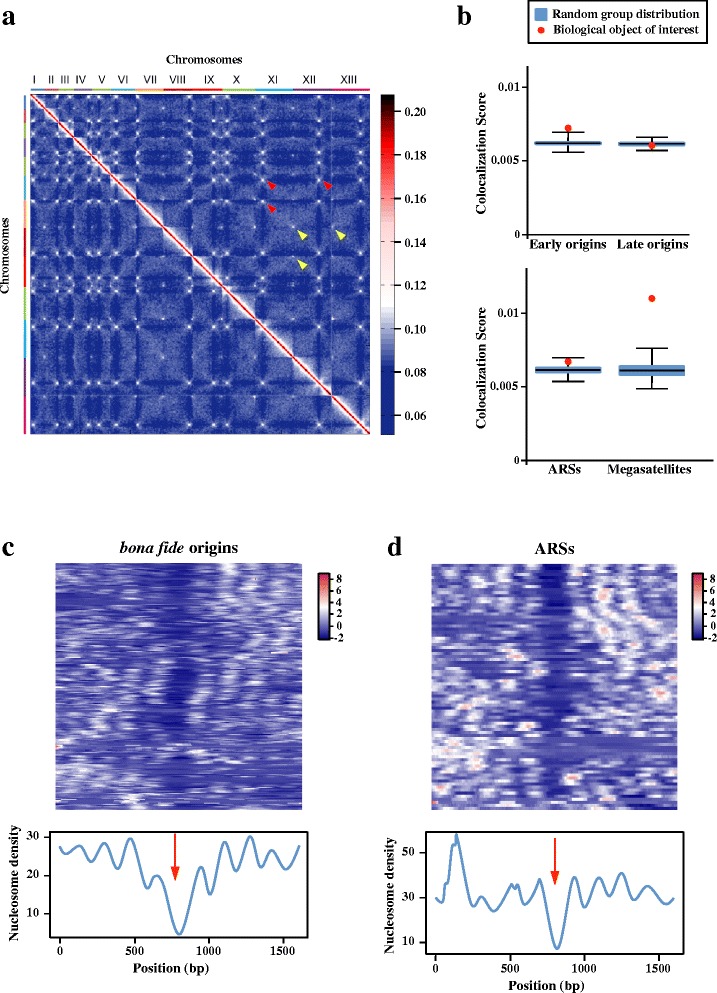
Chromatin organization of *C. glabrata* exponentially growing cells. **a** Normalized genomic contact matrix obtained from an asynchronous growing population. The 13 chromosomes are indicated on the *x axis*. The color scale on the right indicates the frequency of interactions between two regions of the genome (*blue* rare contacts; *white* frequent contacts; *red* very frequent contacts; exponential scale). *Red arrowheads* centromere clustering. *Yellow arrowheads* telomere contacts. **b** Colocalization Score (CS) for DNA regions of interest. The CS is the mean of measured contacts between DNA regions. The boxplots represent the distribution of CS expected by chance, obtained with 1,000 randomized sets of positions (keeping overall chromosomal distribution), whereas the *red dot* corresponds to the CS of each group of interest (Megasatellites, ARSs, early and late origins). Statistical significance of colocalization is attained when the red dot lies above the random group distribution (*p*-value < 0.001). **c** Nucleosomal organization at bona fide origins. Nucleosome signals 800 bp upstream and downstream of bona fide origins were aligned (*top*), the color code representing nucleosome density (*blue* low density; *red* high density). The *bottom curve* represents the average value of nucleosome density. Regular nucleosome spacing is observed, with a large depletion at replication origins (*red arrow*). Note that ACS positions (instead of ARS/origins positions) were used, to increase resolution. **d** Nucleosomal organization at all ARSs. Nucleosome density around all ACS positions were determined and also show a significant depletion. *ACS* ARS consensus sequence, *ARS* autonomously replicating sequence

In *S. cerevisiae*, early replication origins exhibit enriched tridimensional contacts in the nucleus [43]. In order to characterize whether this organization could be reproduced in another ascomycete, a genomic 3C experiment was performed on *C. glabrata*. As previously described for *S. cerevisiae*, the *C. glabrata* contact map exhibits a typical Rabl pattern, with centromeres as well as telomeres presenting highly significant clustering [44] (Fig. 7a). A contact score, reflecting the enrichment in contacts between regions of interest as compared to a null model, was computed for early and late replication origins (Fig. 7b and Additional file 8) [45]. Early replication origins presented significantly enriched contacts, in contrast to late origins, similar to what was observed in *S. cerevisiae* [43]. The association of megasatellites with subtelomeric regions resulted in a clustering effect, but megasatellites in non-subtelomeric regions did not exhibit any enrichment in contacts (data not shown).

## Discussion

In the present work, we identified 253 replication origins and 275 ARSs in the pathogenic yeast *C. glabrata*. These numbers are comparable to those found in other Saccharomycotina yeasts like *S. cerevisiae* (247–332 origins [12, 13] and 337 ARSs), *L. kluyveri* (220 origins [15] and 84 ARSs), and *L. waltii* (195 origins and 183 ARSs [6], or *Taphrinomycotina* yeasts like *S. pombe* (143–387 origins [20], and *S. octosporus* (208 origins [20]), but are significantly lower than in *S. japonicus* (526–542 origins [20]. In other yeast species studied, most replication origins fall within intergenic regions. Here, all the 83 bona fide origins were located in intergenes, confirming the strong bias observed in other yeast species [6, 7, 9].

Only one ARS was previously identified in the *C. glabrata* genome, on chromosome F (286,060–286,210), in strain BG2, and used to build a series of replicating *C. glabrata* plasmids [46]. A replication peak was found on chromosome F at position 286,761, and an ARS was detected at 285,933–286,107 (ARS_F6). When both ARS sequences were aligned, 48 bp were found in common between the two sequences. Right in the middle of these 48 bp an ACS was detected (Fig. 6c). The 17-bp A/T-rich ACS detected here is very similar to the ACS described for *S. cerevisiae* [10, 38], showing that despite the evolutionary distance separating these two yeast species [22], binding specificities of ORC/MCM complexes are probably conserved. This is consistent with a former study showing the high level of conservation of proteins involved in replication between *S. cerevisiae* and *C. glabrata* [47]. Similarly, an A/T-rich consensus was found in *L. kluyveri*, *L. waltii*, *S. paradoxus*, and *S. bayanus* [5, 6, 21], proving that ORC/MCM binding specificities are also conserved over larger evolutionary distances. Interestingly, a yeast species recently shown to be a interspecies hybrid [35] contains two different sets of ARSs. AT-rich ARSs are reminiscent of the *S. cerevisiae* type, whereas GC-rich ARSs seem to be more specifically associated with early origins [9]. Analysis of 69 ARSs cloned from *Kluyveromyces lactis* showed that the ACS was longer (50 bp) in this yeast species [7]. The authors proposed that although *K. lactis* have fewer ARSs than *S. cerevisiae*, the larger size of *K. lactis* ARSs allows them to encompass more features important for replication, making them more efficient than *S. cerevisiae* ARSs. It seems that *C. glabrata*, with its large number of ARSs, is closer to the *S. cerevisiae* paradigm than to *K. lactis*.

### Fragile sites and replication origins

Fragile sites on metaphasic chromosomes in human cells are defined as regions of chromatin constriction or double-strand broken chromatid(s) that occur when cells are grown in the presence of drugs impeding replication (reviewed in [48, 49]). The precise mechanism(s) for fragile site expression is not fully understood. For the common fragile site *FRA3B*, it was shown that fragility in lymphoblastoid cells was correlated to the lack of replication initiation in this 700-kb region, resulting in cells entering mitosis with an incompletely replicated *FRA3B* locus [50]. For *FRAXA*, a rare fragile site involved in the most common cause of hereditary mental retardation, fragile X syndrome, it was recently shown that the large CGG trinucleotide repeat expansion at this locus impairs replication fork progression coming from an origin immediately adjacent to the repeat tract [51]. Hence, the current model postulates that chromosomal fragility is triggered by a paucity of nearby replication origins or by the blocking of a nearby origin by a specific DNA sequence, leading in both cases to mitosis entry with incompletely replicated chromatid(s). In *S. cerevisiae*, it was shown that regions containing replication fork termination sites often contain genetic elements involved in fork pausing (centromeres, tRNA genes, long terminal repeats, etc.) [11]. It was therefore suggested that these late-replicated regions would be favored sites for chromosomal breakage. In *C. glabrata*, eight chromosomal breakpoints that are commonly found between different strains have been precisely mapped by previous authors [30, 31]. Interestingly, chromosomal breakpoints are more often located near late-replicating origins (seven occurrences out of eight breakpoints, Fig. 3d), the only breakpoint near an early-replicating origin being a terminal truncation of chromosome F [31]. This suggests that *C. glabrata* chromosomes are likely to break more often in regions that may be incompletely replicated, owing to the late arrival of forks coming from late-firing origins. That observation will be interesting to confirm when more rearrangement breakpoints are precisely characterized to obtain significant numbers. In *L. kluyveri* and *L. waltii*, no association between synteny breakpoints and replication origins could be found, but the authors did not differentiate between early and late replication origins [15], so it was impossible to compare our results.

### Histone genes and nucleosomes

Highly expressed genes appear to be replicated early during S phase, in yeast as well as in human cells [52]. In *S. cerevisiae*, histone-encoding genes are replicated earlier, on average, than the rest of the genome [12]. Histone expression level is also dependent on replication [53], suggesting that early duplication of histone genes leads to an early doubling of their mRNA levels, ensuring efficient DNA duplication and wrapping around histones of the rest of the genome. Indeed, replication origins near histone genes were found to be highly conserved between *S. cerevisiae* and *L. waltii* [6]. In *S. cerevisiae*, there are two copies of H2A, H2B, H3, and H4 histone genes, whereas there is only one copy of H1 histone gene. In *C. glabrata*, there are also two copies of H2A and H2B histone genes, but there are three copies each of H3 and H4 histone genes. The third copies are named *CgHHT3* and *CgHHF3*, respectively. Our study demonstrated that histone genes are frequently located in early-replicating regions. *CgHTA2*, *CgHTB2*, *CgHHT3*, and *CgHHF3* were located around the chromosome C centromere, and *CgHHT1* and *CgHHF1* were near the chromosome H centromere, all of them early replicating (Fig. 2 and Additional file 9). H1 histone, encoded by *CgHHO1*, was in an early/late region, whereas only two histone genes, *CgHHT2* and *CgHHF2*, were in a late-replicating area. We conclude that histone genes are mainly located in early-replicating regions of the *C. glabrata* genome.

Nucleosomes are known to be depleted in yeast promoter regions. This has been shown for several yeast species, including *C. glabrata* [54]. Using nucleosome positioning data, we found that all 83 bona fide origins were located in nucleosome-depleted regions (Fig. 7c). The same observation was made for the 275 ARSs (Fig. 7d). This confirms that replication origins (and ARSs) are mainly located in promoter regions.

### Revisiting the *C. glabrata* cell cycle

Generation doubling time was determined to be 79–87 min (mean 83 min) in our experimental conditions (SC-Ura medium, 30 °C). Given that S phase itself lasts 20–25 min (Additional file 1), roughly 1 h is left to complete the cell cycle until the next S phase. The observation that unbudded cells, freshly elutriated, already exhibited a 15 % increase in DNA content suggests that the *C. glabrata* G1 phase of the cell cycle must be shorter than the *S. cerevisiae* G1 phase, with early origins probably firing shortly after mitosis exit. This may explain—at least in part—why *C. glabrata* is slightly more resistant to γ irradiation than haploid *S. cerevisiae* cells (but less so than diploids) [47]. Spending less time in G1, *C. glabrata* would have more opportunities to repair lethal double-strand breaks using sister chromatids. This will need to be confirmed by additional dedicated experiments.

## Conclusions

We determined the complete replication program of *C. glabrata* using two complementary approaches, deep-sequencing of synchronized replicating cells and ARS capture. Mapping of genetic elements to replication profiles showed that natural chromosomal fragile sites are clearly located in regions of late replication, suggesting that forks tend to collapse and break more easily in these regions. No correlation could be found between the replication program and megasatellites, suggesting that their formation and propagation might not be directly caused by replication fork initiation or termination. It is striking that the ACS is conserved between *C. glabrata* and *S. cerevisiae*, despite their large evolutionary distance, suggesting that the same exact set of proteins is involved in replication initiation in both yeasts. Finally, clustering of early origins in this yeast genome raises the intriguing question of the conservation of this genomic feature among other ascomycetes.

## Methods

### Centrifugal elutriation

An overnight pre-culture of CBS138 *C. glabrata* cells was performed in 200 ml SC-Ura at 23 °C. The culture was diluted to 6 × 10^7^ cells/ml in 800 ml SC-Ura, and antifoaming agent (Antifoam 204, Sigma ) was added to the culture to limit air bubble formation that could impede elutriation. Use of YPD instead of SC-Ura resulted in a high proportion of cell aggregates, making elutriation impossible. Cells were returned to growth for 2 h at 23 °C. The large elutriation chamber (40 ml volume, JE-5.0 rotor, Beckman) was loaded with ca. 5 × 10^10^ cells, at 23 °C, 3500 rpm, at a flow rate of 20–32 ml/min (increasing flow rate by 2 ml/min every 2 min) in an Avanti J-26×PI elutriation centrifuge (JE-5.0 rotor, Beckman). A peristaltic pump Masterflex (L/S Digital standard drive, LC-07523-47) was used with silicone tubings (Masterflex, diameter 1/8° in., CN-96410-16) for all subsequent loading, washing, and collecting operations. After chamber loading, equilibration was performed for 1 h in 1× PBS buffer, at 3500 rpm, at a flow rate of 32 ml/min. After equilibration, the flow rate was increased by 2 ml/min every 2 min, until the first cells exiting the chamber were detected, at a flow rate of 48 ml/min. From that point, cells were collected in 500 ml fractions, checked under the microscope, and centrifuged in 50 ml sterile polypropylene tubes. Preliminary experiments showed that, in contrast to *S. cerevisiae*, *C. glabrata* enters S phase before the bud may be detected on mother cells. Hence, cell collection was stopped when the first buds were detected on elutriated cells. The flow rate was increased by 2 ml/min every 500 ml fraction, until budded cells became visible under the microscope (Olympus BH-2), at a flow rate of ca. 60 ml/min. Altogether, ca. 8 l of PBS-containing small unbudded yeast cells were collected and centrifuged, for a final amount of 5 × 10^9^ G1 cells (10 % of the initial amount of cells in culture). These cells were resuspended in 500 ml fresh preheated YPD medium and incubated at 30 °C. At this stage it is crucial to use YPD instead of SC-Ura and to incubate at 30 °C instead of 23 °C, otherwise cells re-enter cell cycle asynchronously. Collections were made at ten time points (ca. 5 × 10^8^ cells for sequencing and 5 × 10^7^ cells for FACS analysis), every 5 min after 55 min. For FACS analysis, cells were incubated overnight in 70 % ethanol, washed in 50 mM sodium citrate (pH 7.5), sonicated to remove aggregates, and incubated for 1 h in 50 mM sodium citrate containing 2 μM Sytox Green (Invitrogen) [55]. Cells were loaded on a MACSQuant Analyser (Miltenyi Biotec) to determine relative proportions of cells in the G1 and G2 phase of the cell cycle. The relative amount of DNA at each time point was determined using the formula (G1 cells + 2 × G2 cells)/(G1 cells + G2 cells) [13] (Additional file 1).

### Library preparation for deep sequencing

*C. glabrata* cells collected at six time points were digested with Zymolyase (300 μg, 100 T, Seikagaku, Japan), and total genomic DNA was phenol-extracted and precipitated. Each library was made from 5 μg DNA, sonicated to an average size of 500 bp (Bioruptor, maximum power (H), 30" ON/30" OFF cycles, nine cycles). DNA ends were subsequently repaired with T4 DNA polymerase (15 units, New England BioLabs, Boston, MA, USA) and Klenow DNA polymerase (5 units, New England BioLabs) and phosphorylated with T4 DNA kinase (50 units, New England BioLabs). Repaired DNA was purified on two MinElute columns (Qiagen) and eluted in 16 μl (32 μl final for each library). Addition of a 3' dATP was performed with Klenow DNA polymerase (exo-) (15 units, New England BioLabs) and home-made adapters were ligated with 2 μl T4 DNA ligase (New England BioLabs, high concentration, 2 × 10^6^ units/ml). DNA was size-fractionated on a Pippin Prep (Sage Science) and the fraction containing 400–600 bp DNA fragments was recovered in LoBind microtubes (Eppendorf). Recovered DNA was PCR-amplified with Illumina primers PE1.0 and PE2.0 and Phusion DNA polymerase (1 unit, Thermo Scientific) for nine cycles. Twenty-five PCR reactions were pooled, for each library, and purified on Qiagen purification columns (two columns were used for 25 PCR reactions). Elution was performed in 60 μl (twice 30 μl) and DNA was quantified on a spectrophotometer and on an agarose gel.

### Analysis of Illumina reads and determination of replication origins

Each library was run on a HiSeq 2000 (Illumina) as 50-bp single read sequences. From 112 to 146 millions reads were obtained for each library, corresponding to 466–608× coverages for the *C. glabrata* genome. *FastX_quality_stat* [56] was used to assess read quality. Because read quality was high enough (average quality > 20), no read was discarded at that stage. Repeated sequences, especially megasatellites, were replaced by “G” stretches in the reference sequence (*C. glabrata* complete genome sequence, release 10/09/2008, [57, 58]), before mapping was performed using *BWA* [59]. Reads whose sequence quality was below 20 (Phred quality) were trimmed during mapping. After trimming, reads whose mapping quality was below 20 [60] were discarded (2.6–2.8 % of total reads of each time point), *mpileup* (SAMtools [60]) was run to determine the coverage of each nucleotide, and duplicate reads were removed by *rmdup* (SAMtools [60]). Coverages were finally imported in the *R* package [61].

In order to remove under-representations and over-representations in each library, aberrant coverage values between (coverage median/2) and (coverage median × 2) were filtered. Coverages were corrected for the amount of DNA measured by FACS at each time point (Additional file 1). Normalization was performed by multiplying each coverage value by the ratio (median coverage at time point Tn/median coverage at time point T0).

T_50_ were calculated, for each nucleotide, at each time point between T1 and T6, by plotting coverages at each time point. A four-parameter logistic non-linear regression was used to calculate the inflection point (T_50_) of each sigmoid regression:

$$ F(x)=d+\frac{a-d}{1+{\left(\frac{x}{c}\right)}^b} $$

with a = minimum coverage; b = slope; c = inflection point; d = maximum coverage, x = coverage at time point Tn [62].

Finally, locally weighted scatterplot smoothing [63] was applied to smooth T_50_ curves. Several span values were used, from almost no smoothing (0.0001) to extensive smoothing (0.075), and results were compared with data obtained from ARS capture experiments. This allowed us to empirically determine that a span value equal to 0.04 was the optimum (Additional file 10). Subsequently to smoothing, local maxima were computed to determine peaks corresponding to replication origins [64].

### Fork speeds

Only early replication origins firing before 69 min were taken into consideration. Average ratios were calculated by normalizing sequence coverage at T3 (65') and T4 (70') with T0 coverage. Average rates corresponded to slopes between normalized T3 and T4 mean coverages. The same procedure was applied every 400 bp from the origin, up to 20 kb away, and the corresponding 50 average rates were plotted (Fig. 3b). Linear regressions of fork rates for the first 2 kb (orange line) or 5 kb (red line) are shown. These lines were extrapolated to the baseline, at which no replication was occurring (horizontal blue line). Intersects occured at 10.5 kb and 15.1 kb distance from origins, corresponding to average fork speeds of 2.1–3 kb/min.

### Plasmid construction for ARS identification

The pMEG1 plasmid was built from a pBlueScript II SK(+) backbone (Stratagène) in which the *CgURA3* gene (*CAGL0I03080g*) was cloned. The gene was amplified from CBS138 genomic DNA, using primers CgURA3for (GGAAGATCTTCCTCCTGTAATTACAACAATTCAA) and CgURA3rev (CGGGATCCCGGTTGCCATTACGCCACGCGAGC), amplifying a 1,597-bp fragment containing the *CgURA3* gene plus 500 bp upstream and 300 bp downstream of the open reading frame. This PCR fragment was digested by BglII and BamHI and ligated to pBlueScript digested by BamHI, and dephosphorylated. The resulting pMEG1 plasmid was sequenced and no mutation was found in *CgURA3* or in its flanking regions. A pMEG1-derivative pMEG1s plasmid was built by inserting phosphorylated and annealed SC126 (CCGGGAACGTAGTGACAGGTAC) and SC127 (CTGTCACTACGTTCCCGGGTAC) oligonucleotides into the unique KpnI site of pMEG1. Hence, pMEG1s carries a unique SmaI site surrounded by KpnI sites. Neither pMEG1 nor pMEG1s naturally propagate in *C. glabrata*.

### *C. glabrata* genomic library for ARS identification

Genomic DNA from *C. glabrata* CBS138 strain was isolated as described above. We sonicated 20 μg of DNA to an average size of 200 bp on a Covaris S220 (MicroTUBE AFA Fiber; peak power 175; duty factor 10; cycles/burst 200; 280 s) and fragments of size 150–250 bp were purified from a 3 % MetaPhor gel (Lonza) using Qiaquick columns (Qiagen). Fragmented ends (5 μg) were repaired with T4 DNA polymerase (15 units, New England BioLabs) and Klenow DNA polymerase (5 units, New England BioLabs) and phosphorylated with T4 DNA kinase (50 units, New England BioLabs). Repaired DNA fragments were purified on a Qiaquick column and eluted in 50 μl Tris pH 8. For library construction, 160 ng of the above purified DNA fragments were ligated into the dephosphorylated SmaI site of pMEG1s (1.55 μg) with T4 DNA ligase (2,000 units, New England BioLabs). To maximize cloning efficiency, ligation products (100 μl) were purified on Qiaquick columns and eluted in 50 μl 10 mM Tris pH 8. Ligation products were then used to transform 1 ml of SURE2 competent *Escherichia coli* cells [65, 66]. Colonies were pooled and plasmid DNA was extracted using the alkaline lysis method followed by phenol–chloroform extraction and ethanol precipitation. Approximately 450,000 clones bearing inserts were obtained, corresponding to a total library coverage of about 7× genome size.

### ARS identification and replication competition experiment

To select ARS-containing plasmids, the above library was transformed into the *ura3*∆ auxotrophic *C. glabrata* CBS138-derivative HM100 strain (*ura3*∆*::KAN* [32]) using a modified version of the lithium acetate-heat shock method [67]. Transformed cells were plated onto complete SD medium lacking uracil. After 3 days at 30 °C, 1,558 independent [URA+] clones were obtained in the first experiment and 2,616 clones were obtained in the second experiment. Clones of the first experiment were pooled (G0) and one tenth of this pool was then used for competition experiment. Briefly, cells were diluted into complete liquid SD medium lacking uracil and grown over 50 (G50) and 100 (G100) generations, by sequential dilutions every eight generations. To minimize genetic drift due to bottleneck effect during the course of selection, each diluted culture contained ~5.7 × 10^8^ cells, corresponding to an average representation of 365,000 copies of each ARS-containing plasmid.

Genomic DNAs from the initial pool (G0) and after 50 (G50) and 100 (G100) generations were prepared as described above and then used (5 μl out of 200 μl) to transform 500 μl of SURE2 competent *E. coli* cells. Up to 2.5 × 10^6^ clones were obtained after transformation of each library. Colonies were pooled and plasmidic DNAs were extracted using the alkaline lysis method followed by phenol–chloroform extraction and ethanol precipitation. Inserts from each library (~3 μg) were PCR-amplified with SC139 (GTAAAACGACGGCCAGTGAA) and SC140 (GTAGACAAGAACCCATAGAC) primers and Phusion DNA polymerase (1 unit per reaction, Thermo Scientific) for eight cycles. PCR products were digested with KpnI (100 U, Boehringer Mannheim) and purified from agarose gel using Qiaquick columns. Libraries for deep sequencing were prepared as described above, with the exception that fractionation on Pippin Prep was omitted.

### ARS library sequencing

Paired-end reads were generated on a MiSeq (Illumina). From 332,000 to 1,418,000 reads (250 bp on each side) corresponding to 8–34× coverages, depending on the library, were obtained. Reads were treated like previously (see above). The distribution of read coverage for the initial library (before yeast transformation) is shown in Additional file 6. Contiguous positions covered at least six times, without interruption, were scored as ARSs, independently for each library. Mean coverage of each library was determined as ∑(ARS length × coverage)/genome size.

Given that the ARS library was blunt-ended cloned, it was possible that more than one insert was present in some of the replicating plasmids. To identify double inserts, paired-end reads whose reads mapped on two different chromosomes were computed. Out of the 28 double inserts identified, 25 contained one or more ACS-containing insert. As a complementary experiment, 14 plasmids corresponding to reads in which no ACS could be clearly identified by Illumina mapping were individually amplified and sequenced from both ends. All of them but one corresponded to double inserts and one to a triple insert. In each case, one of the inserts contained the ACS, confirming the previous result. Hence, in most plasmids carrying double (or triple) inserts, at least one of the inserts contains an ACS. As a final proof, seven ACS-containing inserts and eight ACS-less inserts were individually cloned in the same *URA3* plasmid used for library building. All the plasmids containing an ACS were able to replicate in *C. glabrata*, whereas none of those containing an ACS-less fragment could propagate. The seven ARSs that efficiently propagated a plasmid were located 0.46–16.6 kb from the closest replication origin and are highlighted in Additional file 7.

### ACS consensus determination

From ARS coordinates (at G0), sequences were extracted from the reference genome to constitute an ARS database. In order to detect conserved motifs, GIMSAN [68] was run on the ARS library. The null model used by GIMSAN was built from sequences that were not in the ARS database. Motif sizes of 8, 14, or 20 bp were used as input parameters. When the 20-bp size was used, the consensus sequence was not significant beyond 14 bp, therefore a 14-bp consensus was kept. The GIMSAN output file was reformatted in the MEME file format. The 14-bp consensus was subsequently researched in all ARS sequences extended 200 bp upstream and downstream, using MAST [69]. Recalculation of the *S. cerevisiae* ACS was made using the same approach as for *C. glabrata*, using as a library the 337 ARSs identified in the budding yeast genome [70]. Subsequent comparisons of consensus between *C. glabrata* ARSs and bona fide origins showed that this 14-bp consensus could be slightly extended upstream and downstream to a 17-bp consensus that fit with the *S. cerevisiae* extended ARS consensus (Fig. 6a).

### Phylogenetic analyses

All chromosome C proteins were extracted from the Génolevures database [71], and compared to *Naumovozyma castellii*, *Kluyveromyces polysporus*, and *Zygosaccharomyces rouxii* complete proteomes. Gene families containing paralogous genes were removed, so that only orthologous families containing one and only one gene in each of the four yeast species were retained for subsequent comparisons. On chromosome C left arm, 105 genes were found to be in orthologous gene families, whereas 39 orthologous gene families were found on the right arm. In each family, the distance between the *C. glabrata* gene and each of its three orthologues was calculated and a Z-score was determined for this distance. Average Z-scores were slightly higher for genes located on the right arm than for the left arm for all three species (Fig. 4b). However, statistical analysis of Z-score distributions did not show any significant difference between *N. castellii* and *Z. rouxii* gene distances (*t*-test, left arm *p*-value = 0.7971, right arm *p*-value = 0.7043), nor between *N. castellii* and *K. polysporus* (*t*-test, left arm *p*-value = 0.9383, right arm *p*-value = 0.9087).

### Chromosome conformation capture

*C. glabrata* cells (strain CBS138) were recovered from an exponentially growing population (YPD medium). Cells were cross-linked for 10 min with fresh formaldehyde (3 % final concentration), pooled as aliquots of 3 × 10^9^ cells, and stored at −80 °C until use. Aliquots were thawed on ice and resuspended in 6 ml 1× *Dpn*II buffer (New England BioLabs) and 3C libraries were generated as described [72].

The 3C libraries were sheared and processed into Illumina libraries using custom-made versions of the Illumina PE adapters (Paired-End DNA sample Prep Kit, Illumina, PE-930-1001). Fragments of sizes between 400 and 800 bp were purified using a Pippin Prep apparatus (SAGE Science), PCR amplified, and paired-end sequenced on an Illumina platform (HiSeq2000; 2 × 100 bp). After sequencing, PCR duplicates were collapsed using the random tag present on each of the custom-made adapters and reads were aligned using Bowtie 2 in its most sensitive mode against the *C. glabrata* reference genome [73]. An iterative alignment procedure similar to [74] was used. Paired reads were aligned independently. Each mapped read was then assigned to a restriction fragment. Genome-wide contact matrices were built by binning the genome into units of 30 or 60 restriction fragments, resulting in 1351 × 1351 and 679 × 679 contact maps used for the colocalization score (CS) calculation and Fig. 7, respectively. The contact maps were subsequently normalized using the sequential component normalization procedure described in Cournac et al. [45]. This procedure ensures that the sum over the column and lines of the matrix equals 1, which reduces both noise and biases inherent to the protocol. The CS of each feature of interest corresponds to the average number on contacts found in bins which contain the feature. The statistical significance of CS was assessed using a random sampling method [43] (Cournac and Koszul, unpublished). A thousand random sets of bins were generated and the actual CS value was compared with the distribution of CS obtained under the null hypothesis.

### Nucleosome positioning

The following data file from Tsankov *et al.* [54] was used: GSM552914_Cgla_CM_Dec0908_nucCount_100.wig. Density heatmaps of nucleosome occupancies were plotted using the R function heatmap3. In this function, a Z transformation is used to increase pattern contrast between nucleosome-depleted and nucleosome-rich areas. Hierarchical clustering was used to classify sequences.

### Data deposition

Raw Illumina sequences for replication origins, as well as for ARS capture experiments were uploaded at the EBI (accession number: PRJEB8571). Scripts used for the analyses were uploaded in a GitHub repository [75].

## Additional files

Additional file 1:
**DNA content after elutriation.** **A** FACS analyses and determination of DNA content. The first cell collection was made 55 min after T0, because we previously determined that this was the amount of time required for *C. glabrata* cells to re-enter the cell cycle after elutriation. Subsequent collections were made at 5 min intervals. DNA was labeled with Sytox Green [56] and analyzed by FACS (MACS Quant, Miltenyi Biotec) and relative amounts of G1 and G2 cells were calculated using FlowJo. Note that at T0, there is already a detectable amount of cells that have started to replicate their genome, suggesting that replication restarts soon after mitosis, at early origins. For each time point, peaks corresponding respectively to G1 and G2 cells are shown, along with percentages of cells in each phase. Dotted lines represent average intensity values of G1 and G2 peaks. **B** Table summarizing these results. DNA content was calculated using the formula (G1 cells + 2 × G2 cells)/(G1 cells + G2 cells) [13]. The curve plots DNA content according to each time point. Note that time points are spaced at 5 min, except between T0 and T1 (55 min). Genomic DNA from time points shaded in gray were extracted and sequenced. **C** FACS profiles of the first elutriated fraction of two independent experiments (T0). The small peak corresponds to cells that have already started DNA replication, although no bud is visible by visual examination. (PDF 230 kb) Additional file 2:
**Position and firing time of each replication origin, by chromosome.** Coordinates are given according to Genolevures sequence release 10 September 2008 (www.genolevures.org). Bona fide origins are in red (see text). (DOC 154 kb) Additional file 3:
**Plasmid replication efficacy of DNA regions surrounding identified replication origins.** (DOC 40 kb) Additional file 4:
**Additional experiment performed to confirm replication origins.** The time course shown in this experiment was performed as described in “Methods” for the first experiment (Additional file 1). Top: FACS analyses and DNA content are shown. Time points T0 to T6 were sequenced on a MiSeq (Illumina) sequencer and 2.3–4.1 millions reads were obtained (175 bp single reads) for each time point. Bottom: Sequence coverage for time points T2, T3, T4, and T6, for each chromosome (smoothing span: 0.05–0.09, depending on chromosome size). Sequence mapping of two time points (T1 and T5) exhibited too many gaps to allow us to calculate non-linear regressions for each of the 12 millions nucleotides of the *C. glabrata* genome, in order to determine T50 values. However, using T6 and T0 coverage, which correspond respectively to S and G1 phases of the cell cycle, we were able to determine replication peak positions. A comparison of peaks detected in experiment #1 (Fig. 2) and experiment #2 (the present figure) was made in the bottom right table. Out of 83 bona fide origins, 73 were found in experiment #2 (88 %), and 241 replication origins were detected (instead of 253 in experiment #1). The average distance between bona fide replication peaks found in both experiments was 6.9 ± 2.4 kb (95 % confidence interval), consistent with what was deduced from comparisons between ARS positions and replication peaks (Fig. 5a). (PDF 1224 kb) Additional file 5:
**rDNA locus replication.** Top: One tandem repeat unit of the *S. cerevisiae* rDNA locus on chromosome 12 is represented. Coordinates are shown in boxes, according to the Saccharomyces Genome Database (release 19 November 2012). The ARS present in each repeat unit is indicated as a blue diamond. Bottom: Same representation for the *C. glabrata* rDNA locus. Coordinates are shown according to Génolevures database (release 10 September 2008). The two ARSs captured are shown by blue diamonds. (PDF 65 kb) Additional file 6:
**ARS library.** Number of ARSs (y axis) for each coverage (x axis) in the ARS library, before transformation. Coverage was determined by Illumina sequencing (see “Methods”). The average coverage of the library was 9×. Each bar represents 2× coverage. (PDF 98 kb) Additional file 7:
**Positions, coverages, and fitnesses of ARSs.** (XLS 69 kb) Additional file 8:
**Colocalization scores calculated from a second** ***C. glabrata*** **3C matrix [** 77 **] for each genetic element studied.** Using this data set, in addition to early origins, a set of 275 ARSs colocalize. See Fig. 7 for legend. Note that in this experiment, cells were cross-linked for 30 min (instead of 10 min) with fresh formaldehyde (3 % final concentration). (PDF 35 kb) Additional file 9:
**Replication timing of histone genes.** (DOC 49 kb) Additional file 10:
**Effect of smoothing on replication curve shape.** The chromosome G replication curve is represented after smoothing with different span values, from 0.0001 (almost no smoothing) to 0.075 (extensive smoothing). With low levels of smoothing, several peaks or shoulders did not correspond to ARS positions and were clearly artifacts (0.015 span value). This effect was particularly pronounced for small chromosomes. With extensive smoothing (0.075 span value), close replication peaks tended to merge into one single peak, like, for example, the large left-most double (or triple) replication peak. Comparison of different smoothing levels with ARS positions (red dots) led us to use a 0.04 span value for all chromosomes. (PDF 1101 kb)

## Abbreviations

3C: chromosome conformation capture
bp: base pair
ACS: ARS consensus sequence
ARS: autonomously replicating sequence
CS: colocalization score
FACS: fluorescence-activated cell sorting
kb: kilobase pair
nt: nucleotide
SC-Ura: synthetic complete Ura medium
YPD: yeast extract peptone dextrose
