# Supplementary figures and images for: Genome-wide replication landscape of Candida glabrata
Source: BMC Biol. 2015 Sep 2;13:69. doi: 10.1186/s12915-015-0177-6 (PMC4556013; doi:10.1186/s12915-015-0177-6)

**A**

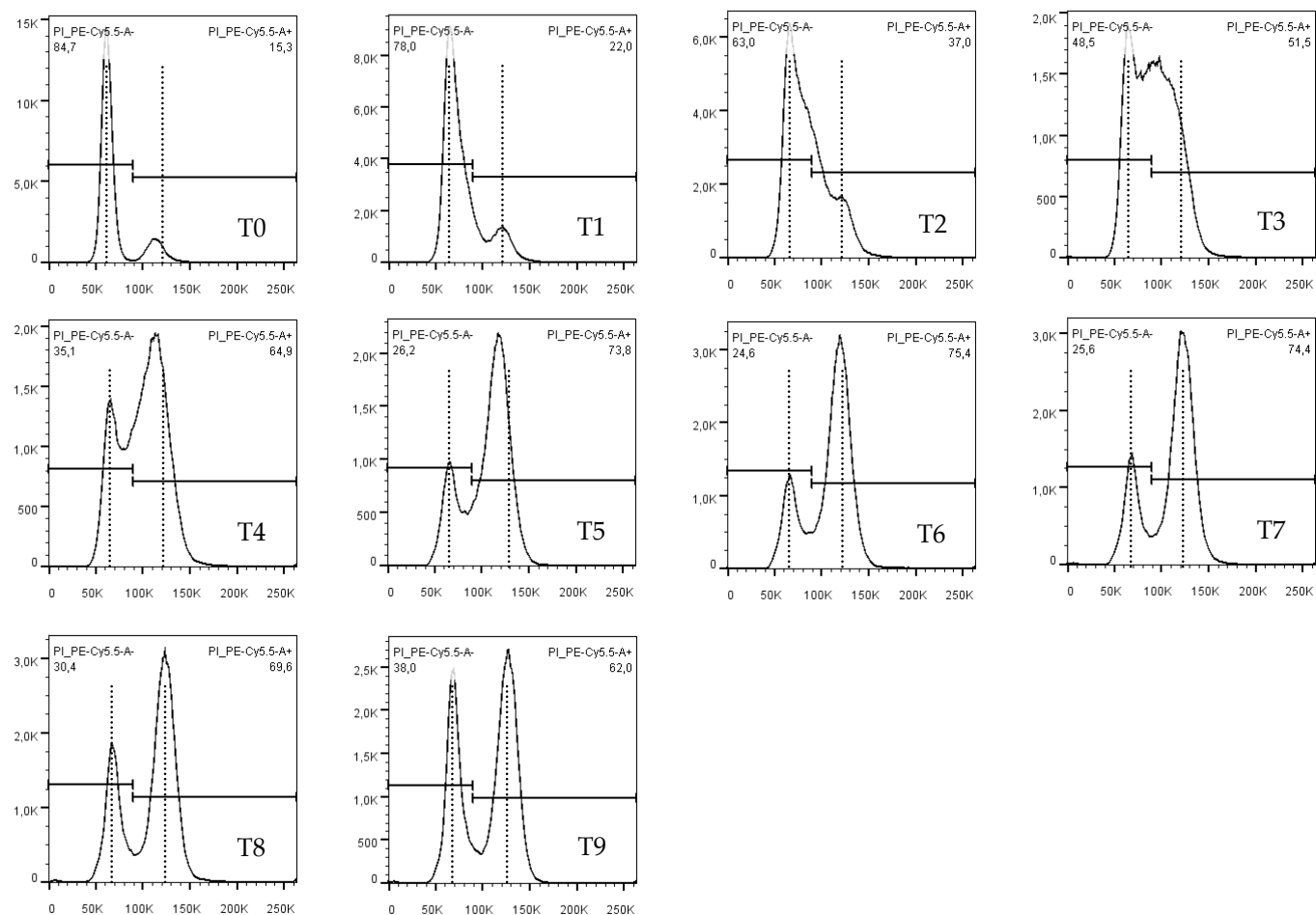

**B** Fraction Time G1 (%) G2 (%) DNA content

| Fraction | Time | G1 (%) | G2 (%) | DNA content |
|----------|------|--------|--------|-------------|
| T0       | 0    | 84.7   | 15.3   | 1.15        |
| T1       | 55'  | 78.0   | 22.0   | 1.22        |
| T2       | 60'  | 63.0   | 37.0   | 1.37        |
| T3       | 65'  | 48.5   | 51.5   | 1.51        |
| T4       | 70'  | 35.1   | 64.9   | 1.65        |
| T5       | 75'  | 26.2   | 73.8   | 1.74        |
| T6       | 80'  | 24.6   | 75.4   | 1.75        |
| T7       | 85'  | 25.6   | 74.4   | 1.74        |
| T8       | 90'  | 30.4   | 69.6   | 1.70        |
| T9       | 95'  | 38.0   | 62.0   | 1.62        |

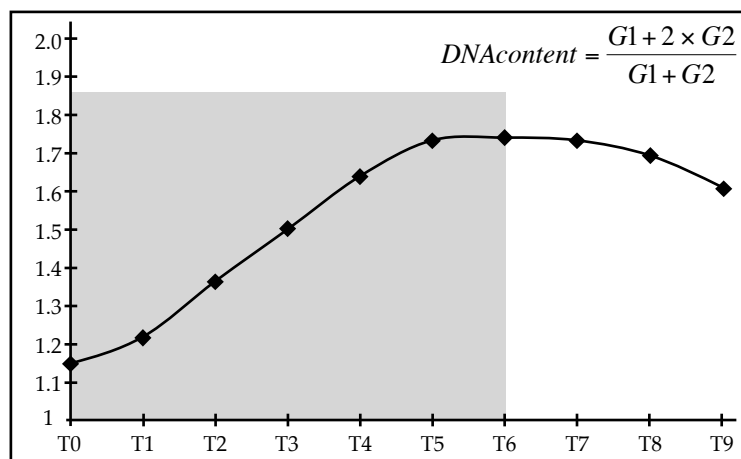

**C**

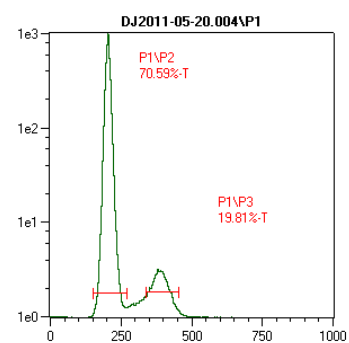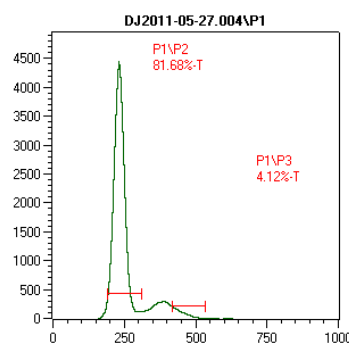

Supplement: Additional file 1: — DNA content after elutriation. A FACS analyses and determination of DNA content. The first cell collection was made 55 min after T0, because we previously determined that this was the amount of time required for C. glabrata cells to re-enter the cell cycle after elutriation. Subsequent collections were made at 5 min intervals. DNA was labeled with Sytox Green [56] and analyzed by FACS (MACS Quant, Miltenyi Biotec) and relative amounts of G1 and G2 cells were calculated using FlowJo. Note that at T0, there is already a detectable amount of cells that have started to replicate their genome, suggesting that replication restarts soon after mitosis, at early origins. For each time point, peaks corresponding respectively to G1 and G2 cells are shown, along with percentages of cells in each phase. Dotted lines represent average intensity values of G1 and G2 peaks. B Table summarizing these results. DNA content was calculated using the formula (G1 cells + 2 × G2 cells)/(G1 cells + G2 cells) [13]. The curve plots DNA content according to each time point. Note that time points are spaced at 5 min, except between T0 and T1 (55 min). Genomic DNA from time points shaded in gray were extracted and sequenced. C FACS profiles of the first elutriated fraction of two independent experiments (T0). The small peak corresponds to cells that have already started DNA replication, although no bud is visible by visual examination. (PDF 230 kb) [file 12915_2015_177_MOESM1_ESM.pdf]

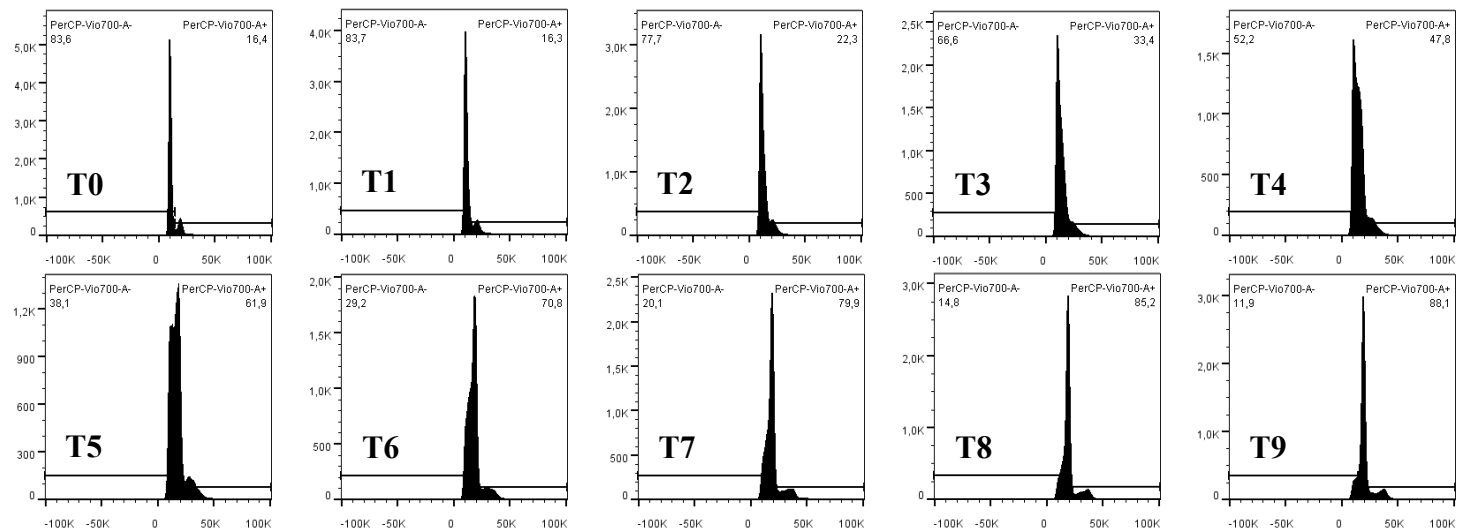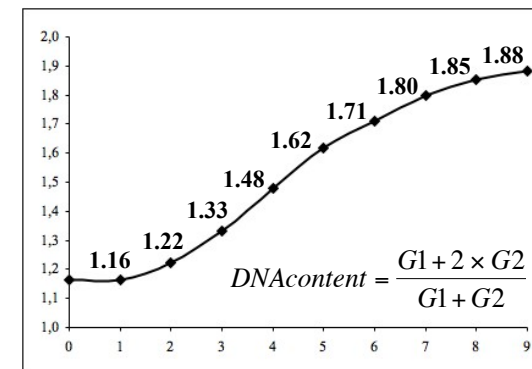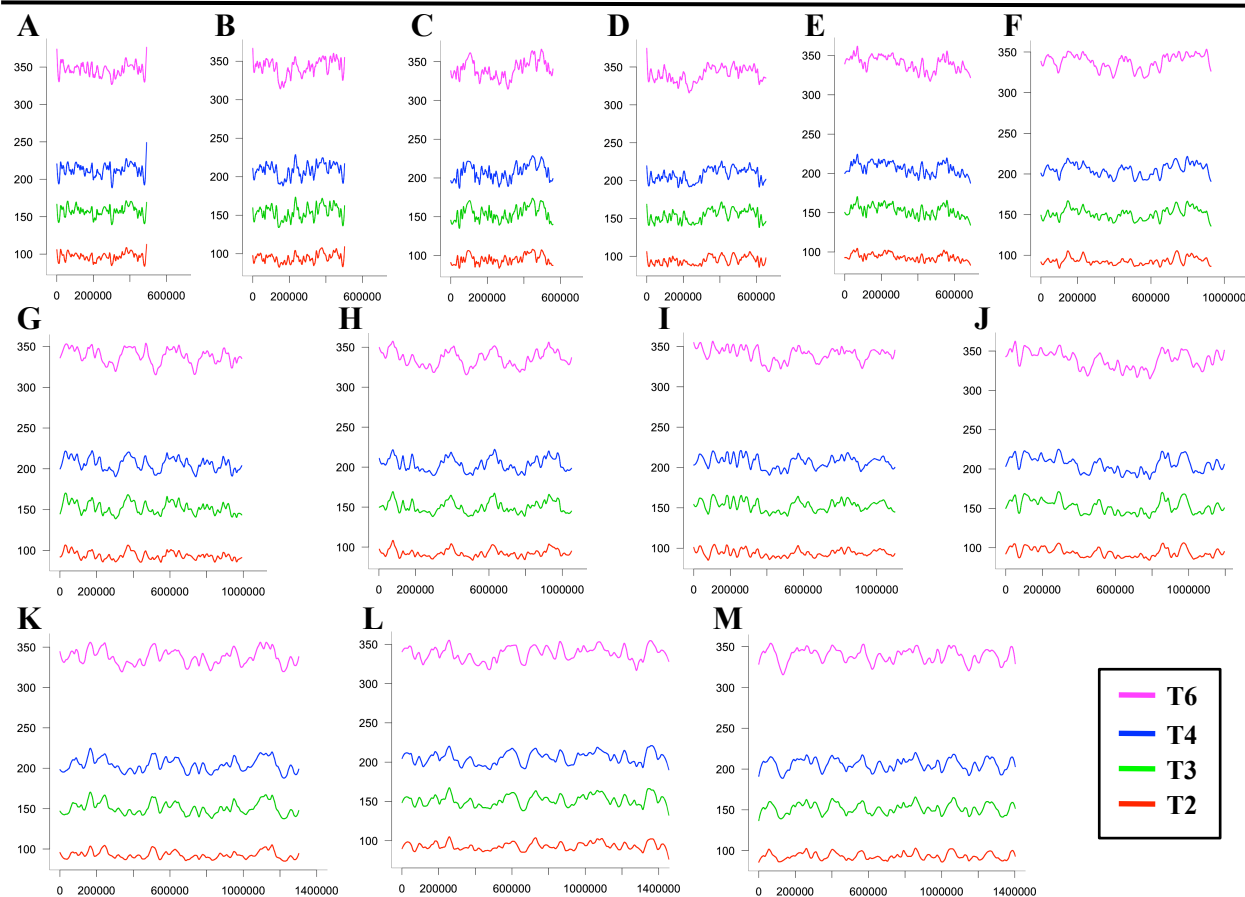

| Chrom.       | Exp #1    |            | Exp#2     |            | Dist. Bona fide (kb) |
|--------------|-----------|------------|-----------|------------|----------------------|
|              | Bona fide | All        | Bona fide | All        |                      |
| A            | 6         | 16         | 4         | 15         | 14.7                 |
| B            | 3         | 17         | 2         | 12         | 3.7                  |
| C            | 3         | 13         | 3         | 10         | 5.0                  |
| D            | 4         | 15         | 4         | 11         | 10.0                 |
| E            | 8         | 16         | 7         | 12         | 10.7                 |
| F            | 7         | 21         | 6         | 21         | 3.6                  |
| G            | 4         | 19         | 3         | 16         | 5.2                  |
| H            | 5         | 20         | 5         | 21         | 3.5                  |
| I            | 8         | 20         | 8         | 30         | 8.6                  |
| J            | 10        | 22         | 10        | 25         | 6.1                  |
| K            | 9         | 26         | 7         | 22         | 7.9                  |
| L            | 11        | 26         | 9         | 23         | 6.5                  |
| M            | 5         | 22         | 5         | 23         | 3.9                  |
| <b>Total</b> | <b>83</b> | <b>253</b> | <b>73</b> | <b>241</b> | <b>6.9</b>           |

Supplement: Additional file 4: — Additional experiment performed to confirm replication origins. The time course shown in this experiment was performed as described in “Methods” for the first experiment (Additional file 1). Top: FACS analyses and DNA content are shown. Time points T0 to T6 were sequenced on a MiSeq (Illumina) sequencer and 2.3–4.1 millions reads were obtained (175 bp single reads) for each time point. Bottom: Sequence coverage for time points T2, T3, T4, and T6, for each chromosome (smoothing span: 0.05–0.09, depending on chromosome size). Sequence mapping of two time points (T1 and T5) exhibited too many gaps to allow us to calculate non-linear regressions for each of the 12 millions nucleotides of the C. glabrata genome, in order to determine T50 values. However, using T6 and T0 coverage, which correspond respectively to S and G1 phases of the cell cycle, we were able to determine replication peak positions. A comparison of peaks detected in experiment #1 (Fig. 2) and experiment #2 (the present figure) was made in the bottom right table. Out of 83 bona fide origins, 73 were found in experiment #2 (88 %), and 241 replication origins were detected (instead of 253 in experiment #1). The average distance between bona fide replication peaks found in both experiments was 6.9 ± 2.4 kb (95 % confidence interval), consistent with what was deduced from comparisons between ARS positions and replication peaks (Fig. 5a). (PDF 1224 kb) [file 12915_2015_177_MOESM4_ESM.pdf]

*S. cerevisiae*

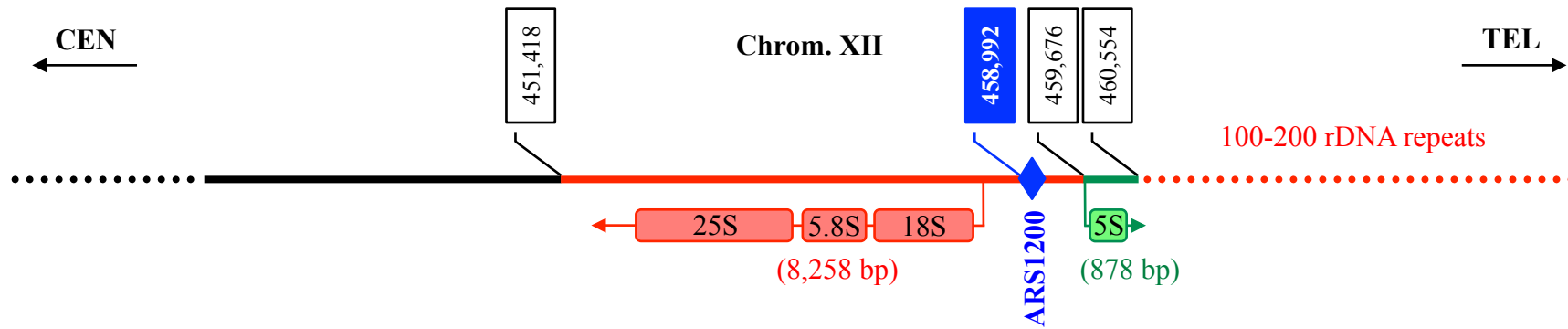

### *C. glabrata*

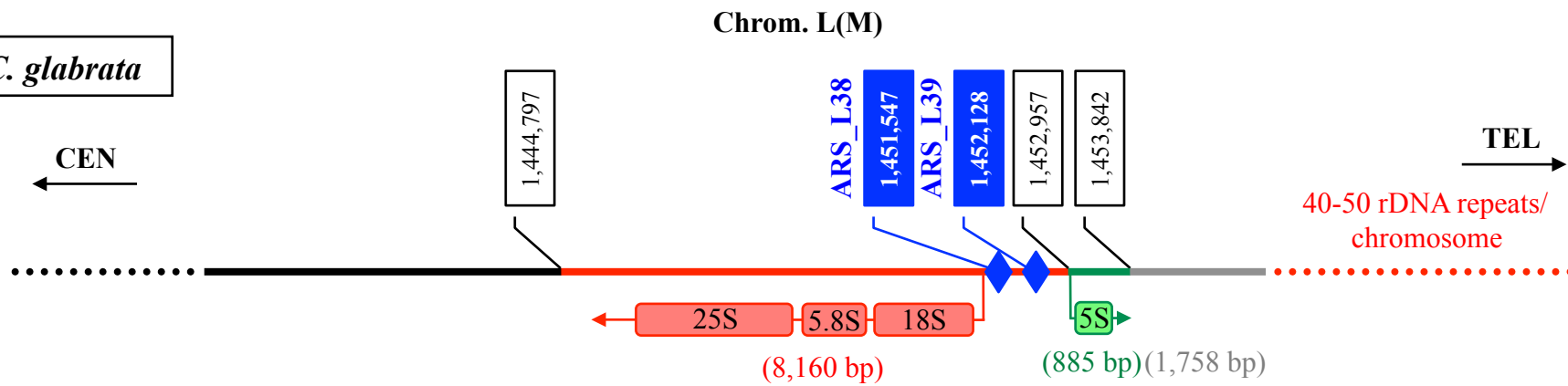

Supplement: Additional file 5: — rDNA locus replication. Top: One tandem repeat unit of the S. cerevisiae rDNA locus on chromosome 12 is represented. Coordinates are shown in boxes, according to the Saccharomyces Genome Database (release 19 November 2012). The ARS present in each repeat unit is indicated as a blue diamond. Bottom: Same representation for the C. glabrata rDNA locus. Coordinates are shown according to Génolevures database (release 10 September 2008). The two ARSs captured are shown by blue diamonds. (PDF 65 kb) [file 12915_2015_177_MOESM5_ESM.pdf]

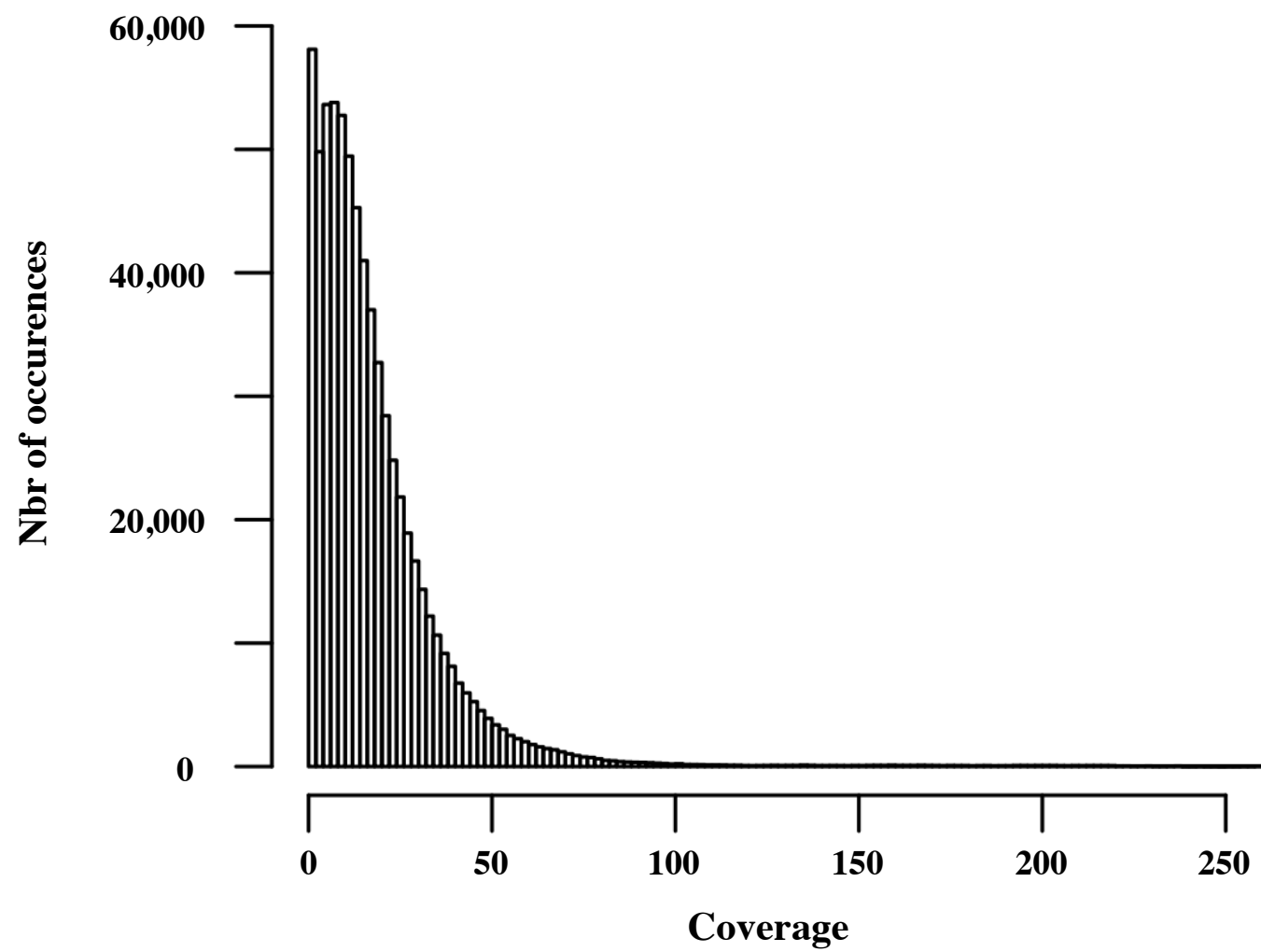

Supplement: Additional file 6: — ARS library. Number of ARSs (y axis) for each coverage (x axis) in the ARS library, before transformation. Coverage was determined by Illumina sequencing (see “Methods”). The average coverage of the library was 9×. Each bar represents 2× coverage. (PDF 98 kb) [file 12915_2015_177_MOESM6_ESM.pdf]

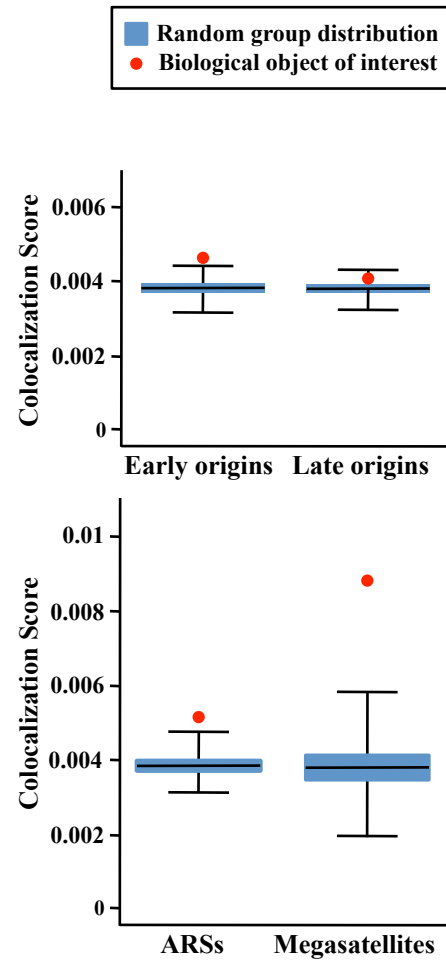

Supplement: Additional file 8: — Colocalization scores calculated from a second C. glabrata 3C matrix [ 77 ] for each genetic element studied. Using this data set, in addition to early origins, a set of 275 ARSs colocalize. See Fig. 7 for legend. Note that in this experiment, cells were cross-linked for 30 min (instead of 10 min) with fresh formaldehyde (3 % final concentration). (PDF 35 kb) [file 12915_2015_177_MOESM8_ESM.pdf]

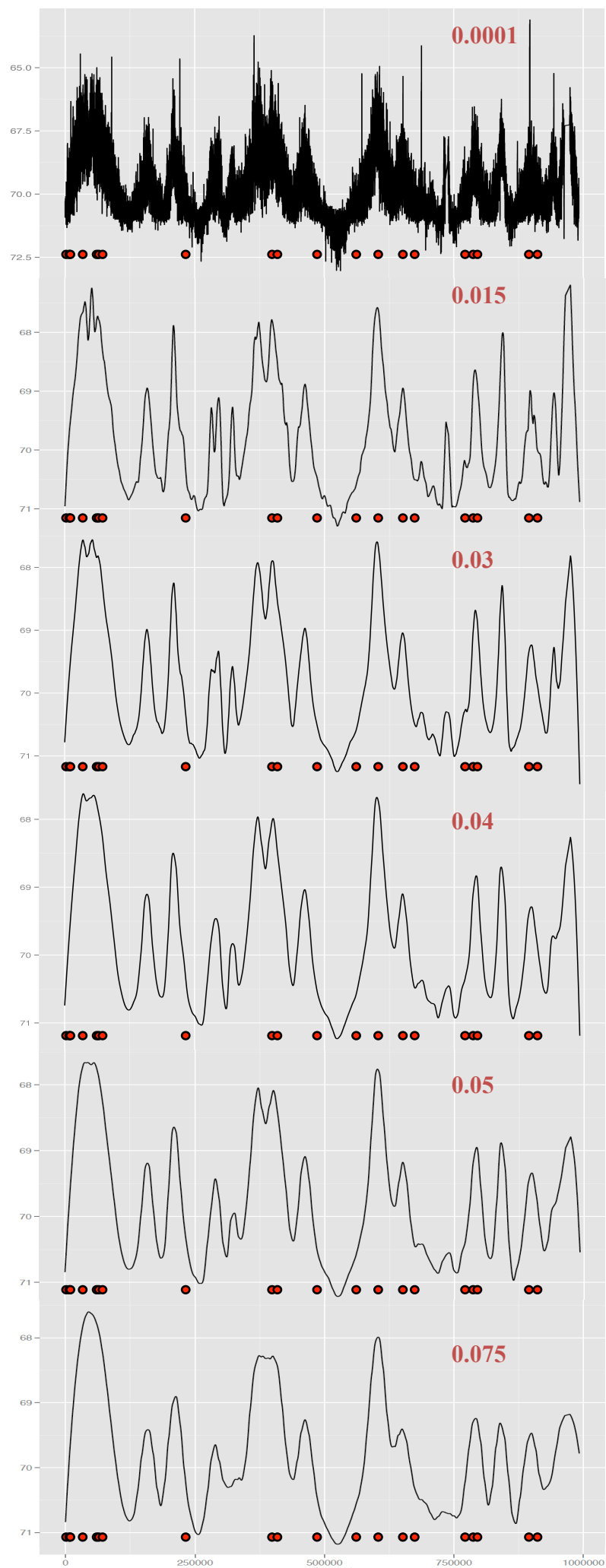

Supplement: Additional file 10: — Effect of smoothing on replication curve shape. The chromosome G replication curve is represented after smoothing with different span values, from 0.0001 (almost no smoothing) to 0.075 (extensive smoothing). With low levels of smoothing, several peaks or shoulders did not correspond to ARS positions and were clearly artifacts (0.015 span value). This effect was particularly pronounced for small chromosomes. With extensive smoothing (0.075 span value), close replication peaks tended to merge into one single peak, like, for example, the large left-most double (or triple) replication peak. Comparison of different smoothing levels with ARS positions (red dots) led us to use a 0.04 span value for all chromosomes. (PDF 1101 kb) [file 12915_2015_177_MOESM10_ESM.pdf]
