# Supplementary material for: Genome-wide replication landscape of Candida glabrata
Source: BMC Biol. 2015 Sep 2;13:69. doi: 10.1186/s12915-015-0177-6 (PMC4556013; doi:10.1186/s12915-015-0177-6)
Supplement: Additional file 2: — Position and firing time of each replication origin, by chromosome. Coordinates are given according to Genolevures sequence release 10 September 2008 (www.genolevures.org). Bona fide origins are in red (see text). (DOC 154 kb) [file 12915_2015_177_MOESM2_ESM.pdf]

A	Time	B	Time	C	Time	D	Time	E	Time	F	Time	G	Time
14509	67.2	25976	67.1	25454	70.5	13087	68.2	46044	67.8	32786	68.8	35825	67.6
39087	69.7	35383	66.9	99753	68.7	85664	68.8	107231	70.6	64500	70.4	49552	67.7
72425	67.8	37530	66.9	173436	70.7	139471	70.6	152665	68.9	101088	69.7	55787	67.6
83232	67.5	94900	68.6	189714	70.8	178315	70.8	182723	69.9	147090	68.1	158415	69.1
157344	70.0	120394	70.5	202244	70.5	201570	70.5	221976	68.6	224868	65.8	208781	68.5
188845	68.2	124062	70.5	250207	70.6	241904	70.9	243845	65.3	287193	70.6	291000	69.5
200903	67.3	139880	70.4	284990	70.7	259266	69.2	292600	70.6	341709	70.7	323314	69.8
246238	67.9	183343	70.8	326500	71.0	295795	69.7	313435	68.3	370858	70.4	371778	68.0
331808	69.5	193109	70.5	380297	68.9	349615	67.6	365225	70.3	398440	70.1	401208	68.0
356979	67.4	208895	70.6	411692	66.9	417644	68.0	383597	70.4	442033	69.0	462112	69.0
370806	68.2	230614	69.5	453283	68.0	444722	67.6	427863	70.2	479174	70.5	600852	67.7
389031	67.9	311144	68.9	502815	68.0	477316	68.1	467864	70.6	514855	70.8	650404	69.1
414487	68.6	331832	70.1	545833	67.6	545334	65.3	490515	70.2	534165	70.3	687741	70.4
459065	66.5	350304	69.5			570118	68.1	543549	65.7	618767	70.4	738789	70.5
473156	68.9	373003	68.0			636307	69.0	600210	70.6	643426	69.7	792365	68.8
475616	68.8	469009	65.8					649141	67.7	677898	69.7	840268	68.7
		485644	68.6							745143	67.4	898410	69.3
										762898	67.8	939942	69.7
										823148	67.8	973645	68.3
										853590	68.9		
										904494	68.0		

H	Time	I	Time	J	Time	K	Time	L	Time	M	Time
19977	67.3	30988	68.0	37068	68.1	62720	68.1	32259	67.6	48465	68.0
76541	67.9	119190	68.0	109880	68.1	170866	69.0	79887	70.6	113931	70.1
105850	68.2	173028	67.1	154951	68.0	259474	66.4	89516	70.5	155263	70.8
142852	70.9	190138	68.0	210302	66.6	305338	70.7	180769	68.2	198151	69.6
184862	70.1	270154	68.1	269001	67.9	313908	70.7	263813	68.0	246251	66.5
224706	70.2	342422	69.6	295586	68.9	364107	69.9	360585	70.5	312386	69.1
282753	69.4	420893	70.6	362797	69.7	396172	70.8	396553	70.3	333631	69.2
315121	70.5	450097	70.0	411570	70.4	437334	70.0	439898	70.6	409354	68.0
385427	67.7	507833	70.5	504233	68.1	505181	68.2	484543	69.6	473782	70.0
416726	68.1	565468	68.4	529836	67.8	534215	69.1	567876	69.0	546198	68.0
499049	69.0	616345	67.8	564542	71.0	577393	68.9	617759	68.3	666212	69.9
553009	69.9	652250	69.8	602670	69.9	618808	67.0	666139	70.4	709627	70.6
620085	68.0	711717	70.4	652436	69.8	664345	70.5	727450	67.9	742248	70.9
712610	70.6	794160	65.8	698758	70.3	670494	70.5	793836	69.4	793541	67.3
752799	70.0	851004	68.0	716267	70.4	723916	69.9	865716	68.2	854511	68.3
793814	70.3	940180	70.6	775049	70.2	756461	69.6	976687	66.7	931029	70.8
816525	70.6	986173	68.2	873411	66.3	836762	69.8	1031348	67.9	1000391	69.3
851633	69.9	1014396	68.1	970820	67.8	905092	69.8	1051801	68.1	1050823	67.2
951703	66.3	1043465	68.1	1042870	69.4	949504	69.6	1097183	68.4	1181439	70.2
1016377	70.6	1086039	68.8	1069237	71.0	993405	71.0	1156445	70.6	1264261	67.5
				1106650	70.2	1022474	69.8	1166213	70.7	1318291	70.0
				1176524	67.8	1111890	67.8	1200858	69.5	1384766	68.4
						1150055	68.0	1241239	70.8		
						1231824	70.1	1251568	70.7		
						1262550	70.4	1360130	68.4		
						1285707	70.3	1413375	68.5		
