# Supplementary material for: Genome-wide replication landscape of Candida glabrata
Source: BMC Biol. 2015 Sep 2;13:69. doi: 10.1186/s12915-015-0177-6 (PMC4556013; doi:10.1186/s12915-015-0177-6)
Supplement: Additional file 3: — Plasmid replication efficacy of DNA regions surrounding identified replication origins. (DOC 40 kb) [file 12915_2015_177_MOESM3_ESM.pdf]

Chrom.	Coordinates	Growth (1)
A	12,640-16,615	-
A	81,188-85,151	-
A	198,260-202,284	-
H	949,328-953,299	-
A	457,005-460,939	+/-
A	354,860-358,777	+/-
C	500,448-504,393	++++
J	209,286-213,150	++++
G (control)	399,360-403,229	++++

(1) ++++ : liquid culture is saturated after 24 hours at 30°C in SC-URA synthetic medium
+/- : liquid culture is not saturated after 4 days at 30°C in SC-URA synthetic medium
- : culture does not grow after 4 days at 30°C in SC-URA synthetic medium
