# Supplementary material for: Genome-wide replication landscape of Candida glabrata
Source: BMC Biol. 2015 Sep 2;13:69. doi: 10.1186/s12915-015-0177-6 (PMC4556013; doi:10.1186/s12915-015-0177-6)
Supplement: Additional file 7: — Positions, coverages, and fitnesses of ARSs. (XLS 69 kb) [file 12915_2015_177_MOESM7_ESM.pdf]

ARS	Start	End	Size	G0	G50	G100	Fitness (1)	Rel. Fitness (%)	Note
ARS_A01	728	1042	314	13			0,0000	0,00	
ARS_A02	17150	17397	247	30	23		0,0060	4,54	(2)
ARS_A03	155026	155370	344	164			0,0000	0,00	
ARS_A04	187390	187631	241	184	6		0,0003	0,19	
ARS_A05	245879	246115	236	35	53	13	0,0167	12,71	
ARS_A06	267062	267228	166						(3)
ARS_A07	325343	325534	191	198			0,0000	0,00	
ARS_A08	359904	360073	169						(3)
ARS_A09	387374	387599	225						(3)
ARS_B01	12536	12754	218	551	1125	797	0,0301	22,90	
ARS_B02	34926	35191	265	810	1340		0,0129	9,80	(2)
ARS_B03	123051	123431	380	117	16		0,0011	0,81	
ARS_B04	289293	289470	177	83			0,0000	0,00	
ARS_B05	323344	323429	85						(3)
ARS_B06	341761	341916	155						(3)
ARS_B07	372964	373178	214	350			0,0000	0,00	
ARS_B08	457055	457317	262	203			0,0000	0,00	
ARS_C01	1415	1652	237	14			0,0000	0,00	
ARS_C02	65130	65365	235	170			0,0000	0,00	
ARS_C03	100125	100450	325	206	103	22	0,0082	6,22	
ARS_C04	107964	108290	326	209	134	21	0,0081	6,19	(2)
ARS_C05	110987	111119	132						(3)
ARS_C06	112625	112798	173	89	7		0,0006	0,47	
ARS_C07	121712	121891	179	424	16		0,0003	0,22	
ARS_C08	206436	206601	165						(3)
ARS_C09	235774	235880	106						(3)
ARS_C10	284246	284414	168	6			0,0000	0,00	
ARS_C11	347988	348269	281						(3)
ARS_C12	405822	406042	220						(3)
ARS_C13	440941	441145	204						(3)
ARS_C14	462628	462815	187						(3)
ARS_C15	500988	501185	197						(3)
ARS_D01	147	482	335	2275	274	28	0,0030	2,27	
ARS_D02	2429	2681	252	584	115	12	0,0036	2,76	
ARS_D03	13693	13840	147	204	672	374	0,0368	28,00	
ARS_D04	49047	49288	241						(3)
ARS_D05	57929	58063	134	41	6		0,0011	0,87	
ARS_D06	140068	140283	215	6			0,0000	0,00	
ARS_D07	188006	188235	229	618	20		0,0003	0,19	
ARS_D08	202061	202327	266	691	275	49	0,0067	5,08	
ARS_D09	204923	204958	35	6			0,0000	0,00	
ARS_D10	269225	269273	48	6			0,0000	0,00	
ARS_D11	289312	289723	411	618	689	58	0,0104	7,89	
ARS_D12	316707	316830	123						(3)
ARS_D13	349416	349690	274	148	8		0,0004	0,32	
ARS_D14	450130	450273	143	158	262	53	0,0170	12,91	
ARS_D15	450329	450477	148	167			0,0000	0,00	

ARS_E01	604	847	243	158	99	15	0,0079	6,02	
ARS_E02	11903	12309	406	821	59	6	0,0026	1,98	
ARS_E03	47248	47599	351	175	2186	761	0,1042	79,28	(2)
ARS_E04	65482	65710	228						(3)
ARS_E05	70682	70883	201						(3)
ARS_E06	106300	106542	242	17			0,0000	0,00	
ARS_E07	137866	138083	217						(3)
ARS_E08	159089	159270	181						(3)
ARS_E09	182978	183126	148	95	101	16	0,0115	8,71	
ARS_E10	219116	219362	246	110	149	11	0,0120	9,15	
ARS_E11	242609	243008	399	1500	1461	187	0,0101	7,72	
ARS_E12	257581	257756	175	38			0,0000	0,00	
ARS_E13	307592	307737	145	15			0,0000	0,00	
ARS_E14	314840	315076	236	817	190	39	0,0059	4,51	
ARS_E15	415667	415814	147	180			0,0000	0,00	
ARS_E16	415824	415860	36						(3)
ARS_E17	544579	544777	198	1729			0,0000	0,00	
ARS_E18	584956	585088	132	76			0,0000	0,00	
ARS_E19	601321	601620	299	53			0,0000	0,00	
ARS_E20	641254	641491	237						(3)
ARS_F01	354	684	330	1028	154	8	0,0022	1,68	
ARS_F02	116533	116718	185	841	765	96	0,0096	7,30	(2)
ARS_F03	135703	135955	252						(3)
ARS_F04	148224	148455	231	135	12		0,0007	0,53	
ARS_F05	199792	200024	232						(3)
ARS_F06	285933	286107	174	843	37		0,0003	0,26	
ARS_F07	340282	340484	202	1213	528	30	0,0045	3,44	
ARS_F08	409161	409421	260						(3)
ARS_F09	418302	418503	201	198	100	15	0,0069	5,28	
ARS_F10	444368	444600	232	23	46	9	0,0195	14,83	
ARS_F11	471720	472032	312	2557	5571	3090	0,0281	21,36	
ARS_F12	484879	485066	187	4615	1078	85	0,0034	2,59	
ARS_F13	537355	537524	169	177			0,0000	0,00	
ARS_F14	677186	677471	285	1584	1155	250	0,0100	7,62	
ARS_F15	822648	822950	302	1868			0,0000	0,00	
ARS_F16	854809	855109	300	17			0,0000	0,00	
ARS_F17	897332	897576	244	855			0,0000	0,00	
ARS_G01	357	742	385	137	45		0,0026	1,95	
ARS_G02	1369	1434	65						(3)
ARS_G03	1879	2048	169						(3)
ARS_G04	31031	31262	231	82			0,0000	0,00	
ARS_G05	62319	62573	254	46			0,0000	0,00	
ARS_G06	62665	62880	215	124	9		0,0006	0,43	
ARS_G07	67145	67372	227	412	134	16	0,0049	3,75	
ARS_G08	230023	230264	241	14			0,0000	0,00	
ARS_G09	399355	399565	210	6195	3197	444	0,0068	5,17	
ARS_G10	405701	405933	232						(3)
ARS_G11	486165	486299	134	123	43	13	0,0088	6,68	

ARS_G12	561571	561818	247	81			0,0000	0,00	
ARS_G13	603212	603476	264						(3)
ARS_G14	650828	651049	221	382	66		0,0013	1,02	
ARS_G15	674141	674321	180	353	190	48	0,0093	7,04	
ARS_G16	770672	770894	222	3756	9766	4670	0,0298	22,69	
ARS_G17	785395	785608	213						(3)
ARS_G18	794070	794296	226	601	3683	524	0,0506	38,46	
ARS_G19	894937	895101	164						(3)
ARS_G20	910591	910857	266	4231			0,0000	0,00	
ARS_H01	523	900	377	1010	134	25	0,0048	3,63	
ARS_H02	1971	2202	231	81	36	6	0,0068	5,17	
ARS_H03	56932	57138	206						(3)
ARS_H04	77005	77193	188						(3)
ARS_H05	133707	133782	75						(3)
ARS_H06	155793	155988	195						(3)
ARS_H07	160138	160346	208						(3)
ARS_H08	185806	186045	239	1175	348	21	0,0035	2,67	
ARS_H09	232683	232952	269						(3)
ARS_H10	332230	332382	152						(3)
ARS_H11	340778	340990	212	2029	873	23	0,0039	2,95	
ARS_H12	390488	390720	232	25			0,0000	0,00	
ARS_H13	433274	433562	288	4364	9246	4514	0,0263	19,99	(2)
ARS_H14	441847	442082	235	129			0,0000	0,00	
ARS_H15	554718	554974	256	123			0,0000	0,00	
ARS_H16	657244	657486	242	76	30		0,0031	2,34	
ARS_H17	747381	747604	223	26			0,0000	0,00	
ARS_H18	815067	815256	189						(3)
ARS_H19	851157	851387	230	279			0,0000	0,00	
ARS_H20	860698	860880	182						(3)
ARS_H21	866599	866750	151						(3)
ARS_H22	936610	936791	181						(3)
ARS_H23	955216	955483	267						(3)
ARS_I01	136	484	348	274	29		0,0008	0,63	
ARS_I02	1530	1854	324	565	139	13	0,0038	2,88	
ARS_I03	55901	56035	134	78	50	7	0,0078	5,93	
ARS_I04	117605	117885	280	362	552	77	0,0147	11,16	
ARS_I05	162598	162718	120						(3)
ARS_I06	217237	217515	278	110	64	7	0,0067	5,11	
ARS_I08	343823	344156	333	1216	6371	5300	0,0575	43,72	
ARS_I09	369606	369818	212						(3)
ARS_I10	448861	449108	247	9664	17798	11309	0,0271	20,60	
ARS_I11	454656	454702	46						(3)
ARS_I12	500573	500769	196	18			0,0000	0,00	
ARS_I13	566761	567059	298	7954	27520	25550	0,0455	34,65	
ARS_I14	621669	621878	209						(3)
ARS_I15	656431	656626	195	424	132	7	0,0035	2,65	
ARS_I16	694507	694546	39						(3)
ARS_I17	695342	695437	95						(3)

ARS_I18	710099	710255	156						(3)
ARS_I19	712420	712692	272						(3)
ARS_I20	732483	732663	180	150	35		0,0018	1,38	
ARS_I21	761805	762080	275	2528	394	51	0,0038	2,90	
ARS_I22	875628	875824	196						(3)
ARS_I23	912650	912863	213						(3)
ARS_I24	969268	969607	339	1749	429	27	0,0032	2,41	
ARS_I25	970173	970353	180						(3)
ARS_I26	987994	988168	174	365			0,0000	0,00	
ARS_I27	1017317	1017586	269	823			0,0000	0,00	
ARS_I28	1058463	1058649	186	16			0,0000	0,00	
ARS_I29	1088418	1088483	65						(3)
ARS_J01	611	1012	401	385	2708	761	0,0604	45,94	
ARS_J02	23712	23991	279	1136	283	25	0,0037	2,82	
ARS_J03	40508	40792	284	318	316	66	0,0119	9,07	(2)
ARS_J04	101576	101622	46						(3)
ARS_J05	108242	108414	172						(3)
ARS_J06	109843	110097	254	1629	1533	180	0,0097	7,36	
ARS_J07	180039	180242	203						(3)
ARS_J08	269155	269420	265	7219	2842	534	0,0068	5,20	
ARS_J09	296120	296373	253	19			0,0000	0,00	
ARS_J10	363547	363754	207	1721	98		0,0004	0,34	
ARS_J11	364504	364706	202						(3)
ARS_J12	378150	378341	191						(3)
ARS_J13	408164	408342	178						(3)
ARS_J14	422210	422394	184	419	95	8	0,0035	2,63	
ARS_J15	425590	425736	146	14			0,0000	0,00	
ARS_J16	474981	475222	241	6			0,0000	0,00	
ARS_J17	497892	498175	283	10			0,0000	0,00	
ARS_J18	501735	501985	250	54			0,0000	0,00	
ARS_J19	533901	534125	224	222	38		0,0013	1,01	
ARS_J20	608717	609004	287	3609	1545	122	0,0049	3,74	
ARS_J21	696519	696741	222	103			0,0000	0,00	
ARS_J22	742878	743069	191	525	58	6	0,0029	2,23	
ARS_J23	837627	837833	206	2091	1071	322	0,0100	7,62	
ARS_J24	838188	838377	189						(3)
ARS_J25	917218	917353	135	196	523	303	0,0324	24,64	
ARS_J26	970222	970527	305	306	3104	958	0,0851	64,78	
ARS_J27	1034354	1034547	193						(3)
ARS_J28	1040044	1040323	279						(3)
ARS_J29	1107856	1108003	147	158	6		0,0003	0,22	
ARS_J30	1178879	1179372	493	447			0,0000	0,00	
ARS_J31	1192438	1192687	249	8			0,0000	0,00	
ARS_K01	332	670	338	380	36	11	0,0069	5,22	
ARS_K02	1332	1403	71						(3)
ARS_K03	2754	2888	134	8			0,0000	0,00	
ARS_K04	37364	37681	317	351	653	16	0,0150	11,39	
ARS_K05	38364	38536	172						(3)

ARS_K06	82651	82798	147	37	522	563	0,1314	100,00	
ARS_K07	185408	185568	160						(3)
ARS_K08	231858	232043	185	315	152	27	0,0073	5,57	
ARS_K09	233551	233813	262	176	151	38	0,0117	8,92	
ARS_K10	243729	243892	163	863	286	50	0,0061	4,63	
ARS_K11	254565	254757	192	149	33		0,0017	1,31	
ARS_K12	314216	314451	235						(3)
ARS_K13	391166	391372	206	1945	1865	270	0,0104	7,89	
ARS_K14	504798	505044	246	246	11		0,0003	0,26	
ARS_K15	527143	527443	300	62			0,0000	0,00	
ARS_K16	531039	531231	192						(3)
ARS_K17	621116	621423	307	2901	1191	321	0,0086	6,54	
ARS_K18	641689	641904	215	19			0,0000	0,00	
ARS_K19	670153	670366	213						(3)
ARS_K20	762570	762664	94						(3)
ARS_K21	778936	779137	201	62	8		0,0010	0,76	
ARS_K22	840218	840489	271	497	168	25	0,0056	4,27	
ARS_K23	846558	846820	262	185	65	14	0,0071	5,37	
ARS_K24	867044	867099	55						(3)
ARS_K25	874216	874428	212						(3)
ARS_K26	904496	904803	307	1254	1511	794	0,0199	15,15	
ARS_K27	949155	949400	245	1139	38		0,0003	0,20	
ARS_K28	1023113	1023296	183	461	6		0,0001	0,08	
ARS_K29	1073943	1074241	298	7			0,0000	0,00	
ARS_K30	1117755	1117940	185						(3)
ARS_K31	1152867	1153097	230	4672	23654		0,0394	29,98	
ARS_K32	1205630	1205814	184						(3)
ARS_K33	1265257	1265509	252	1113	233		0,0016	1,24	
ARS_L01	117	346	229	67	40	8	0,0087	6,59	
ARS_L02	7530	7732	202	15			0,0000	0,00	
ARS_L03	53064	53355	291	533	378	85	0,0100	7,63	
ARS_L04	110194	110391	197						(3)
ARS_L05	147420	147608	188	29			0,0000	0,00	
ARS_L06	181029	181178	149	8			0,0000	0,00	
ARS_L07	215376	215581	205						(3)
ARS_L08	290761	290990	229	45			0,0000	0,00	
ARS_L09	359511	359818	307	5009	22775	16204	0,0497	37,78	
ARS_L10	391684	391937	253						(3)
ARS_L11	399143	399260	117	90			0,0000	0,00	
ARS_L12	436918	437086	168	38			0,0000	0,00	
ARS_L13	499466	499530	64						(3)
ARS_L14	599314	599498	184						(3)
ARS_L15	615031	615300	269	231	581	75	0,0222	16,86	
ARS_L16	672315	672559	244	1076	210	15	0,0030	2,25	
ARS_L17	725573	725932	359	5909	14476	11897	0,0355	27,05	
ARS_L18	736150	736350	200						(3)
ARS_L19	785388	785578	190	48			0,0000	0,00	
ARS_L20	792198	792430	232	58			0,0000	0,00	

ARS_L21	880790	880976	186	108			0,0000	0,00	
ARS_L22	979093	979358	265	59			0,0000	0,00	
ARS_L23	1008170	1008334	164						(3)
ARS_L24	1028144	1028353	209	84	11		0,0010	0,78	
ARS_L25	1053029	1053200	171						(3)
ARS_L26	1094972	1095210	238	1860	2011	842	0,0168	12,79	
ARS_L27	1160754	1161027	273	985	25		0,0002	0,15	
ARS_L28	1185417	1185615	198						(3)
ARS_L29	1191606	1191773	167						(3)
ARS_L30	1198964	1199342	378	21			0,0000	0,00	
ARS_L31	1201270	1201440	170						(3)
ARS_L32	1208379	1208526	147	9			0,0000	0,00	
ARS_L33	1218774	1218908	134	8			0,0000	0,00	
ARS_L34	1254647	1254881	234						(3)
ARS_L35	1328143	1328300	157						(3)
ARS_L36	1408051	1408168	117						(3)
ARS_L37	1431666	1431842	176						(3)
ARS_L38	1451547	1451657	110						(3)
ARS_L39	1452128	1452450	322	2630			0,0000	0,00	
ARS_M01	8095	8328	233	34	37		0,0085	6,44	
ARS_M02	100947	101135	188						(3)
ARS_M03	229441	229676	235	159			0,0000	0,00	
ARS_M04	309383	309788	405	15			0,0000	0,00	
ARS_M05	452482	452725	243	308	9		0,0002	0,17	
ARS_M06	483607	483991	384	184	54		0,0023	1,74	
ARS_M07	524356	524566	210	99			0,0000	0,00	
ARS_M08	595467	595793	326	1808	1571	240	0,0098	7,48	
ARS_M09	667515	667662	147	80			0,0000	0,00	
ARS_M10	707826	708053	227						(3)
ARS_M11	811421	811485	64	10			0,0000	0,00	
ARS_M12	829455	829672	217						(3)
ARS_M13	853506	853752	246	847	181	10	0,0028	2,11	
ARS_M14	977894	978127	233						(3)
ARS_M15	1183183	1183507	324	966			0,0000	0,00	
ARS_M16	1199193	1199423	230						(3)
ARS_M17	1272920	1273121	201	78			0,0000	0,00	

(1) Fitness: mean fitness
(2) ARS was individually cloned and shown to propagate a plasmid (see M&M)
(3) ARS was identified during the second screen and was not tested in competition with other ARSs
G0, G50 and G100 columns refer to sequence corrected coverage of ARSs tested for fitness at the respective time points
