# Supplementary material for: Genome-wide replication landscape of Candida glabrata
Source: BMC Biol. 2015 Sep 2;13:69. doi: 10.1186/s12915-015-0177-6 (PMC4556013; doi:10.1186/s12915-015-0177-6)
Supplement: Additional file 9: — Replication timing of histone genes. (DOC 49 kb) [file 12915_2015_177_MOESM9_ESM.pdf]

**Histone**	**Gene name**	**CDS ***	**Coordinates**	**Distance to origin**	**T50**	**Timing**
H2A	*CgHTA1*	CAGL0K11440g	1,112,191-1,112,586	498 nt	67.6	Early
H2A	*CgHTA2*	CAGL0C04411g	426,015-426,410	14,520 nt	67.2	Early
H2B	*CgHTB1*	CAGL0K11462g	1,114,096-1,114,491	2,404 nt	66.6	Early
H2B	*CgHTB2*	CAGL0C04389g	424,507-424,896	13,010 nt	68.2	Early
H3	*CgHHT1*	CAGL0H09856g	962,788-963,198	11,290 nt	68.5	Early
H3	*CgHHT2*	CAGL0M06655g	684,606-685,016	18,599 nt	70.4	Late
H3	*CgHHT3*	CAGL0C04114g	404,740-405,150	6,747 nt	68.2	Early
H4	*CgHHF1*	CAGL0H09834g	961,286-961,597	9,739 nt	68.8	Early
H4	*CgHHF2*	CAGL0M06677g	685,761-686,072	19,704 nt	70.2	Late
H4	*CgHHF3*	CAGL0C04136g	406,014-406,325	5,522 nt	67.9	Early
H1	*CgHHO1*	CAGL0M02783g	314,946-315,635	2,904 nt	69.1	Early/Late

* Assignation of a *C. glabrata* CDS to a gene was based on synteny, since histone genes belong to well-conserved paralogous families. Note the two extra *C. glabrata* H3 and H4 genes, as compared to *S. cerevisiae* (*CgHHT3* and *CgHHF3*).
